# Long‐term hospitalisation rates among 5‐year survivors of Hodgkin lymphoma in adolescence or young adulthood: A nationwide cohort study

**DOI:** 10.1002/ijc.30655

**Published:** 2017-03-14

**Authors:** Kathrine Rugbjerg, Maja Maraldo, Marianne C. Aznar, David J. Cutter, Sarah C. Darby, Lena Specht, Jørgen H. Olsen

**Affiliations:** ^1^ Department of Survivorship UnitDanish Cancer Society Research CenterStrandboulevarden 49Copenhagen2100Denmark; ^2^ Department of OncologyRigshospitalet, University of CopenhagenBlegdamsvej 9Copenhagen2100Denmark; ^3^ Nuffield Department of Population HealthUniversity of OxfordRichard Doll Building, Old Road CampusOxfordOX3 7LFUnited Kingdom; ^4^ Department of Oncology, Oxford Cancer and Haematology CentreOxford University Hospitals NHS Foundation TrustOld RoadOxfordOX3 7LEUnited Kingdom; ^5^ Department of HaematologyRigshospitalet, University of CopenhagenBlegdamsvej 9Copenhagen2100Denmark; ^6^ Danish Cancer Society Research CenterStrandboulevarden 49Copenhagen2100Denmark

**Keywords:** Hodgkin lymphoma, cancer survivor, adolescents and young adults, hospitalisations, physical diseases

## Abstract

In the present study, we report on the full range of physical diseases acquired by survivors of Hodgkin lymphoma diagnosed in adolescence or young adulthood. In a Danish nationwide population‐based cohort study, 1,768 five‐year survivors of Hodgkin lymphoma diagnosed at ages 15–39 years during 1943–2004 and 228,447 comparison subjects matched to survivors on age and year of birth were included. Hospital discharge diagnoses and bed‐days during 1977–2010 were obtained from the Danish Patient Register for 145 specific disease categories gathered in 14 main diagnostic groups. The analysis was conducted separately on three subcohorts of survivors, that is, survivors diagnosed 1943–1976 for whom we had no information on rehospitalisation for Hodgkin lymphoma and survivors diagnosed 1977–2004, split into a subcohort with no expected relapses and a subcohort for whom a rehospitalisation for Hodgkin lymphoma indicated a relapse. The overall standardised hospitalisation rate ratios (RRs) were 2.0 [95% confidence interval (CI), 1.9–2.1], 1.5 (1.4–1.6) and 2.9 (2.6–3.1) respectively, and the corresponding RRs for bed‐days were 3.5 (3.4–3.5), 1.8 (1.8–1.9) and 10.4 (10.3–10.6). Highest RRs were seen for nonmalignant haematological conditions (RR: 2.6; 3.1 and 9.7), malignant neoplasms (RR: 3.2; 2.5 and 4.7) and all infections combined (RR: 2.5; 2.2 and 5.3). Survivors of Hodgkin lymphoma in adolescence or young adulthood are at increased risk for a wide range of diseases that require hospitalisation. The risk depends on calendar period of treatment and on whether the survivors were rehospitalised for Hodgkin lymphoma, and thus likely had a relapse.

## Abstract 2

**What's new?**

Excellent survival rates for Hodgkin lymphoma have allowed increasing numbers of patients to enjoy a normal, disease‐free life expectancy. Nonetheless, for some, premature death remains a concern, owing to the risk of late effects, including second malignant neoplasms, from curative therapy. The authors of the present study quantified the burden of new diagnoses in Hodgkin lymphoma survivors, showing that among 1,768 five‐year survivors diagnosed in adolescence or young adulthood from 1943 to 2004, a significant proportion later suffered conditions requiring hospitalisation. Malignant neoplasms, infections and circulatory diseases accounted for the majority of new diagnoses.

The age‐specific incidence of Hodgkin lymphoma shows a bimodal distribution, with a first peak in adolescence and young adulthood and a second peak after the age of 55 years.^1^ As Hodgkin lymphoma is one of the few cancers with a peak in incidence at ages 15–39 years, this cancer is of special interest in adolescent and young adult oncology.^2^ Hodgkin lymphoma diagnosed during adolescence or young adulthood is usually of the nodular sclerosis subtype, in contrast to cases diagnosed at older ages,^3^ and the biology of cancer in adolescents and young adults in general has been suggested to be different from that of cancers in children and older people.^4^, ^5^ Studies of patients in this specific age range are, therefore, important.^6^, ^7^

Hodgkin lymphoma is a cancer with excellent survival. Recent five‐year relative survival in Sweden was 96% for patients whose cancer was diagnosed when they were 18 to 29‐year old, and 95% for those aged 30–39 years.^8^ The treatment of Hodgkin lymphoma may, however, carry a lifelong risk of late effects.^9^, ^10^ Selected late effects in survivors of Hodgkin lymphoma diagnosed during adolescence or young adulthood have been studied, including second malignant neoplasms,^11^ cardiovascular disease^12^ and diabetes.^13^

In a previous publication, we reported on the risk of hospitalisation among survivors of adolescent and young adult cancer in general.^14^ In the present nationwide study, comprising 1,768 five‐year survivors of Hodgkin lymphoma diagnosed at the age of 15–39 years and 228,447 comparison subjects, we provide detailed information on their long‐term relative and absolute excess risks of all types of physical diseases requiring inpatient care.

## Material and Methods

### Hodgkin lymphoma survivor and comparison cohorts

**Figure 1:**
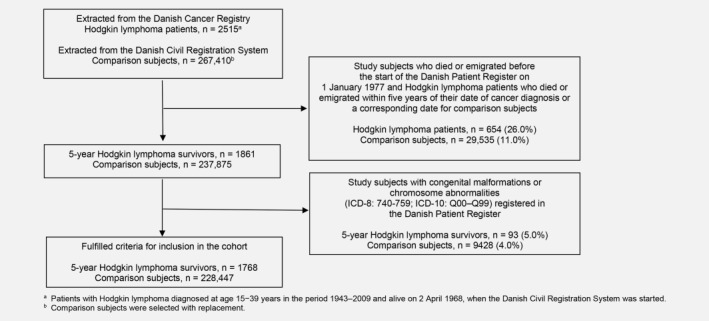
Flow chart showing exclusions from the cohort of adolescent or young adult Hodgkin lymphoma patients and exclusions from the population comparison cohort.

All 2,515 individuals diagnosed with Hodgkin lymphoma (International Classification of Diseases [ICD]‐7: 201; ICD‐O, third edition: 9650/3–9667/3; ICD‐10: C81) as a first primary cancer (apart from nonmelanoma skin cancer), diagnosed at age 15–39 years, notified to the Danish Cancer Registry in the period January 1, 1943 to December 31, 2009, and who were alive on April 2, 1968 (start of the Danish civil registration system) were identified.^15^, ^16^ For each cancer patient, five population‐based comparison subjects were selected randomly from the civil registration system, who were alive without cancer on the date of diagnosis of the corresponding cancer patient (in the following, referred to as the index date) and of the same sex and year of birth. Information on migration and vital status during follow‐up was obtained from the civil registration system. We excluded those who had died or emigrated within five years of the date of cancer diagnosis, or an equivalent lag period for the comparison subjects, and those who had died or emigrated before the start of the Patient Register on January 1, 1977. These exclusions resulted in a cohort of 1,861 five‐year survivors of Hodgkin lymphoma and 237,875 population comparison subjects (Fig. 1).

### Hospital admissions for physical diseases

The Danish National Patient Register holds information on all hospital admissions for nonpsychiatric illnesses in Denmark since January 1, 1977.^17^ Registration is mandatory, and diagnostic information is submitted electronically by treating physicians. Each admission to hospital initiates a record which includes the personal identification number of the patient, dates of admission and discharge, a primary discharge diagnosis and supplementary diagnoses coded according to ICD‐8 until 1993 and according to ICD‐10 thereafter.

Details of cohort members were linked to the Patient Register, and a full hospital history with discharge diagnoses and numbers of bed‐days spent in hospital was established for each person recorded as having had a hospital admission. Individuals who had ever been hospitalised for a congenital malformation or chromosome abnormality (ICD‐8, 740 − 759, ICD‐10, Q00 − Q99) were excluded, as we judged that these disorders might confound any causal association between cancer treatment and chronic disorders. Thus, 1,768 five‐year survivors of Hodgkin lymphoma in adolescence or young adulthood and 228,447 population comparison subjects were left for risk analysis (Fig. 1).

To characterize and quantify the burden of diseases requiring hospitalisation in detail, we grouped the hospital discharge diagnoses into 145 specific disease categories (see Supporting Information Table S1). As the Patient Register does not reliably distinguish hospitalisation for relapse of a primary cancer from hospitalisation for a second primary cancer, we obtained information from the Danish Cancer Registry on second primary cancers among survivors and on first primary cancers among comparison subjects.

In order to take the effect of treatment for relapse of Hodgkin lymphoma into account in the analyses, the cohort of Hodgkin lymphoma survivors was split into the following three subcohorts: (1) survivors diagnosed during 1943–1976 for whom we did not have complete information on rehospitalisations for Hodgkin lymphoma, since the Patient Register started in 1977 (early mixed subcohort; *n* = 494); (2) survivors diagnosed during 1977–2004 for whom we cannot identify any rehospitalisations in a department of oncology or haematology with a primary diagnosis of Hodgkin lymphoma either at least a year after the initial Hodgkin lymphoma diagnosis, or six months to one year after the initial diagnosis in combination with a date of death within ten years from diagnosis (primary treatment only subcohort; *n* = 887), (3) survivors diagnosed during 1977–2004 for whom there is a registration of a rehospitalisation for Hodgkin lymphoma as defined above (assumed relapse subcohort; *n* = 387; Table 1). We emphasize that the 1977–2004 subcohort stratification is based entirely on the available inpatient information.

**Table 1:** Characteristics of 1,768 five‐year survivors of adolescent or young adult Hodgkin lymphoma diagnosed in Denmark, 1943–2004

**Characteristic**	**Both sexes (%)**	**Men (%)**	**Women (%)**
**Total**	1,768 (100)	1,014 (57.4)	754 (42.6)
**Number of person–years**	27,310 (100)	15,300 (56.0)	12,010 (44.0)
**Age at Hodgkin lymphoma diagnosis (years)**			
15 − 19	282 (16.0)	171 (16.9)	111 (14.7)
20 − 24	415 (23.5)	224 (22.1)	191 (25.3)
25 − 29	448 (25.3)	246 (24.3)	202 (26.8)
30 − 34	335 (18.9)	194 (19.1)	141 (18.7)
35 − 39	288 (16.3)	179 (17.7)	109 (14.5)
**Subcohorts of Hodgkin lymphoma survivors**			
Diagnosed in 1943 − 1976:			
Early mixed^a^	494 (27.9)	270 (26.6)	224 (29.7)
Diagnosed in 1977 − 2004:			
Primary treatment only^b^	887 (50.2)	498 (49.1)	389 (51.6)
Assumed relapse^c^	387 (21.9)	246 (24.3)	141 (18.7)
**Years since Hodgkin lymphoma diagnosis**			
5–9	1,768 (100)	1,014 (100)	754 (100)
10–19	1,470 (83.1)	840 (82.8)	630 (83.6)
20–29	879 (49.7)	492 (48.5)	387 (51.3)
30–39	412 (23.3)	214 (21.1)	198 (26.3)
40–49	115 (6.5)	57 (5.6)	58 (7.7)
50–59	21 (1.2)	13 (1.3)	8 (1.1)
**Attained age (years)** d			
20–29	620 (35.1)	355 (35.0)	265 (35.1)
30–39	1,328 (75.1)	731 (72.1)	577 (76.5)
40–49	1,314 (74.3)	760 (75.0)	554 (73.5)
50–59	763 (43.2)	421 (41.5)	342 (45.4)
60–69	345 (19.5)	184 (18.1)	161 (21.4)
70–79	78 (4.4)	41 (4.0)	37 (4.9)
**Type of censoring**			
End of follow‐up (December 31, 2010)	1,233 (69.7)	690 (68.0)	543 (72.0)
Death	514 (29.1)	314 (31.0)	200 (26.5)
Emigration	21 (1.2)	10 (1.0)	11 (1.5)

a No information on rehospitalisations available.

b No rehospitalisation with Hodgkin lymphoma as primary diagnosis in an oncological or haematological department one year or more after the first Hodgkin lymphoma diagnosis or, if rehospitalised for Hodgkin lymphoma 6 months to one year after the first Hodgkin lymphoma diagnosis, did not die within 10 years after the first Hodgkin lymphoma diagnosis.

c Rehospitalisation with Hodgkin lymphoma as primary diagnosis in an oncologic or haematologic department one year or more after the first Hodgkin lymphoma diagnosis, or rehospitalisation 6 months to one year after the first Hodgkin lymphoma diagnosis and deceased within 10 years of the first Hodgkin lymphoma diagnosis.

d Number at entry into category.

In our analysis, we did not include diagnoses indicating symptoms and ill‐defined diseases, or injuries and violence, as these were regarded as too nonspecific and primarily due to external causes, respectively. Neither did we include the sections on mental disorders and pregnancy‐related diseases and complications, as these conditions require special consideration and will be addressed in separate publications.

### Statistical analysis

Follow‐up of study subjects began five years after diagnosis of Hodgkin lymphoma (or after the index date for comparison subjects) or on January 1, 1977 (when the Danish Patient Register was initiated) if this was later. Follow‐up ended on the earliest of date of death, date of emigration or December 31, 2010, which was the date of the latest update of the Patient Register. Only the primary diagnosis, i.e. the main reason for hospitalisation for each inpatient admission, was considered in the analyses.

For analyses of new diagnoses, only the first hospital admission for each of the 145 specific disease categories was considered but, for analyses of the number of bed‐days spent in hospital, all hospital admissions for the disease category were included. For each disease category, the observed number of new diagnoses among all the survivors of Hodgkin lymphoma was divided by the expected number derived from the appropriate sex‐, age‐ and calendar period‐specific diagnosis rates for the comparison subjects to obtain the rate ratio for new diagnoses (RR diagnoses). The corresponding 95% confidence interval (CI) was obtained assuming a Poisson distribution. Rate ratios for bed‐days spent in hospital (RR bed‐days) were obtained in a similar fashion. Absolute excess rates per 10,000 person‐years at risk for new diagnoses and bed‐days (AER diagnoses & AER bed‐days) were derived as the difference between the observed and expected rates for each disease category, and 95% CIs were obtained, again assuming a Poisson distribution.

The observed and expected numbers for the 145 specific disease categories were also summed to obtain RRs and AERs for broader diagnostic groups, including 14 main diagnostic groups based on the Chapters of the ICD. In the ICD a considerable number of infections are classified with diseases of the organ they affect, rather than in the chapter specifically headed ‘Infections’. Therefore, we included an additional broad disease grouping labelled ‘All infections’ that brought together all diagnoses indicating an infection, irrespective of their position in the ICD.

For 94% of patients included in the cohort, the Cancer Registry record specified the intended use of radiotherapy as part of first‐line treatment only on a yes/no level. For survivors included in the primary treatment only subcohort, we specified for risk estimates in patients with (*n* = 338) and without intended use of radiotherapy (*n* = 494). The Danish Cancer Registry does not contain reliable information on treatment with chemotherapy.

All statistical analyses were performed with SAS software version 9.3.

## Results

By the end of the follow‐up period, 514 (29%) of the 1,768 five‐year survivors of Hodgkin lymphoma and 41,012 (18%) of the 228,447 comparison subjects had died. The survivors were followed in the Patient Register for a total of 27,310 person‐years (median 14.4 years, range, 0.1 to 34.0 years). 879 (50%) survivors were followed up for at least 20 years after diagnosis, and 412 (23%) for at least 30 years. Characteristics of the Hodgkin lymphoma survivor group are shown in Table 1.

### Total numbers of new diagnoses and bed‐days

During follow‐up, the 494 five‐year survivors included in the early mixed subcohort had a total of 1,519 new diagnoses belonging to one of the 145 specific disease categories listed in Supporting Information Table S1, whereas only 758 new diagnoses were expected, resulting in a significantly increased overall RR of 2.0. The corresponding RRs for survivors diagnosed in 1977–2004 were 1.5 and 2.9, respectively, for those without and with readmission to hospital for Hodgkin lymphoma (Table 2). The absolute excess rates (AERs) for new diagnoses were 682 per 10,000 person‐years in the early mixed subcohort and 217 and 855 per 10,000 person‐years, respectively in the 1977–2004 primary treatment only and the assumed relapse subcohorts.

**Table 2:** Rate ratios (RRs) and absolute excess rates (AERs) with corresponding confidence intervals (CIs) for the three subcohorts of five‐year survivors of Hodgkin lymphoma diagnosed in adolescence or young adulthood; estimates are given for the total, gender and attained age

	Survivors diagnosed with Hodgkin lymphoma 1943–1976			Survivors of Hodgkin lymphoma diagnosed 1977–2004					
	Early mixed subcohort^a^			Primary treatment only subcohort^b^			Assumed relapse subcohort^c^		
	No. of survivors	RR (95% CI)	AER^e^ (95% CI)	No. of survivors	RR (95% CI)	AER^e^ (95% CI)	No. of survivors	RR (95% CI)	AER^e^ (95% CI)
**NEW DIAGNOSES** d									
**Total**	494	2.0 (1.9–2.1)	682 (614–751)	887	1.5 (1.4–1.6)	217 (168–265)	387	2.9 (2.6–3.1)	855 (751–960)
**Gender**									
Men	270	2.1 (2.0–2.3)	744 (649–839)	498	1.6 (1.5–1.8)	265 (200–329)	246	3.1 (2.8–3.4)	872 (742–1.003)
Women	224	1.9 (1.7–2.0)	612 (513–711)	389	1.3 (1.2–1.4)	156 (83–229)	141	2.6 (2.3–2.9)	826 (653–999)
**Attained age** f **(years)**									
20–29	139	2.0 (1.4–2.8)	351 (110–592)	330	1.3 (1.0–1.7)	90 (−23–203)	151	2.7 (2.1–3.6)	537 (305–770)
30–39	347	1.6 (1.4–1.9)	259 (150–367)	691	1.5 (1.3–1.7)	179 (105–252)	290	2.6 (2.2–3.0)	588 (437–738)
40–49	413	1.8 (1.6–2.0)	403 (300–506)	626	1.2 (1.1–1.3)	93 (20–167)	275	3.0 (2.6–3.4)	962 (782–1,142)
50–59	372	2.4 (2.2–2.6)	954 (810–1,097)	280	1.7 (1.5–2.0)	499 (332–667)	111	3.1 (2.6–3.7)	1,455 (1,090–1,819)
60–69	249	2.3 (2.1–2.5)	1,373 (1,126–1,620)	75	2.4 (1.9–3.0)	1,515 (937–2,093)	21	2.9 (1.9–4.4)	2,018 (693–3,342)
70–79	75	1.6 (1.3–1.9)	1,028 (509–1,547)	3	–	–	–	–	–
**BED‐DAYS**									
**Total**	494	3.5 (3.4–3.5)	20,520 (20,205–23,836)	887	1.8 (1.8–1.9)	3,384 (3,226–3,542)	387	10.4 (10.3–10.6)	37,070 (36,489–37,652)
**Gender**									
Men	270	3.7 (3.7–3.8)	21,948 (21,507–22,389)	498	2.1 (2.0–2.2)	4,299 (4,077–4,521)	246	10.1 (9.9–10.3)	34,826 (34,113–35,539)
Women	224	3.2 (3.1–3.2)	18,899 (18,448–19,350)	389	1.5 (1.5–1.6)	2,217 (1,995–2,438)	141	11.0 (10.8–11.2)	40,848 (39,850–41,845)
**Attained age** f **(years)**									
20–29	139	8.1 (7.6–8.5)	22,193 (20,762–23,625)	330	1.4 (1.3–1.6)	787 (496–1,078)	151	23.3 (22.5–24.2)	47,017 (45,256–48,777)
30–39	347	5.5 (5.4–5.7)	19,603 (18,958–20,247)	691	2.1 (2.0–2.2)	3,111 (2,872–3,350)	290	14.9 (14.5–15.2)	39,799 (38,800–40,797)
40–49	413	3.7 (3.6–3.8)	16,843 (16,333–17,354)	626	1.5 (1.4–1.6)	2,090 (1,850–2,331)	275	9.7 (9.5–10.0)	37,384 (36,418–38,351)
50–59	372	3.8 (3.7–3.9)	24,534 (23,887–25,181)	280	2.0 (1.9–2.1)	6,717 (6,154–7,280)	111	4.0 (3.8–4.2)	19,866 (18,582–21,149)
60–69	249	2.6 (2.6–2.7)	23,679 (22,705–24,652)	75	2.7 (2.5–2.9)	18,495 (16,544–20,446)	21	3.8 (3.4–4.3)	31,008 (26,128–35,887)
70–79	75	1.5 (1.4–1.6)	12,364 (10,495–14,232)	3	–	–	–	–	–

a No information on rehospitalisations available.

b No rehospitalisation with Hodgkin lymphoma as primary diagnosis in an oncological or haematological department one year or more after the first Hodgkin lymphoma diagnosis or, if rehospitalised for Hodgkin lymphoma 6 months to one year after the first Hodgkin lymphoma diagnosis, did not die within 10 years after the first Hodgkin lymphoma diagnosis.

c Rehospitalisation with Hodgkin lymphoma as primary diagnosis in an oncologic or haematologic department one year or more after the first Hodgkin lymphoma diagnosis, or rehospitalisation 6 months to one year after the first Hodgkin lymphoma diagnosis and deceased within 10 years of the first Hodgkin lymphoma diagnosis.

d Hospitalisations for different specific disease categories; see Supporting Information Table S2 and the Methods section for details.

e Absolute excess rate per 10,000 person‐years; the rates are standardised for age, sex and calendar periods.

f Age at entry into category.

For all three subcohorts, the AERs for new diagnoses tended to increase with increasing attained age, while there was little such trend for the RRs (Table 2; Supporting Information Fig. S1, panels A–C). The AERs as well as the RRs for bed‐days varied markedly between the three subcohorts of survivors, with the highest estimates in the 1977–2004 assumed relapse subcohort (Table 2). Table 2 and Supporting Information Figure S1, panel D–F gives the bed‐days by attained age.

### Specific diseases and diagnostic groups

**Figure 2:**
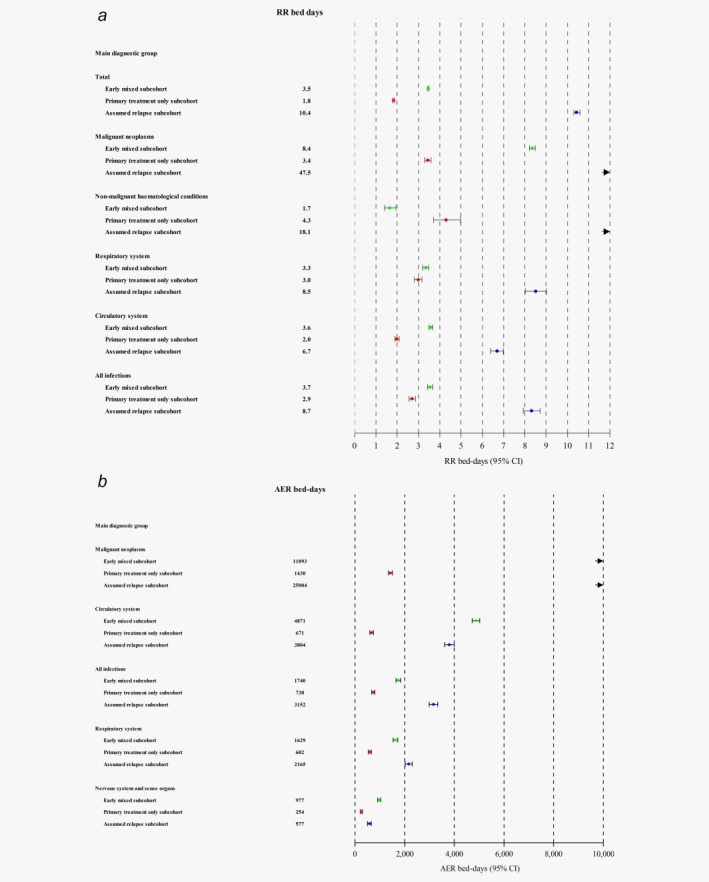
(*a*) Rate ratios (RRs) for diagnoses for selected main diagnostic groups with estimates for each of the three subcohorts. (*b*) Absolute excess rates (AERs) for diagnoses for selected main diagnostic groups per 10,000 person‐years for each of the three subcohorts. The total AERs (not included in the plot) were:‐ for those diagnosed 1943–1976 (i.e., early mixed subcohort): 682, 95% CI, 614–751; for those diagnosed 1977–2004 and with no rehospitalisations (i.e., primary treatment only subcohort): 217, 95% CI, 168–265; for those diagnosed 1977–2004 and with rehospitalisations (i.e., assumed relapse subcohort): 855, 95% CI, 751–960. [Color figure can be viewed at wileyonlinelibrary.com]

The extent of the proportional increases in the rate of new diagnoses varied substantially between the 14 main diagnostic groups, with the largest values seen for nonmalignant haematological conditions, malignant neoplasms, all infections combined, diseases of the respiratory system and diseases of the circulatory system. Rate ratios for these main diagnostic groups are shown in Figure 2, panel A for each of the three subcohorts.

The AERs for new diagnoses revealed a somewhat different pattern. The largest AERs were for diseases of the circulatory system, which accounted for between 23% and 38% of all excess new diagnoses, followed by malignant neoplasms, diseases of the respiratory system and diseases of the digestive organs, while nonmalignant haematological conditions accounted for <5% of the excess new diagnoses (Table 3 and Fig. 2, panel B). As many of the disease entities of the respiratory system are infections, all infections combined was a major cause of hospitalisation accounting for between 14% and 30% of the excess diagnoses in the respective subcohorts. Diseases of the circulatory system, all infections combined and malignant neoplasms explained together between 61% and 78% of the excess inpatient disease pattern among survivors (Table 3).

**Table 3:** Absolute excess rates (AERs) with corresponding 95% confidence intervals (CIs) and percentage of total AER for each of the 14 main diagnostic groups for survivors of Hodgkin lymphoma diagnosed during 1943–1976 and for survivors diagnosed during 1977–2004 with primary treatment only or with assumed relapse

	**Survivors of Hodgkin lymphoma** **diagnosed 1943–1976**		**Survivors of Hodgkin lymphoma** **diagnosed 1977–2004**			
	**Early mixed subcohort** a		**Primary treatment only subcohort** b		**Assumed relapse subcohort** c	
**Main diagnostic group**	**AER** d **(95% CI)**	**% of total AER**	**AER** d **(95% CI)**	**% of total AER**	**AER** d **(95% CI)**	**% of total AER**
Circulatory system	259 (221–297)	38.0	66 (44–88)	30.4	201 (153–249)	23.5
Malignant neoplasms	122 (97–147)	17.9	39 (24–54)	18.0	86 (56–116)	10.1
Respiratory system	95 (72–118)	13.9	42 (25–59)	19.4	179 (137–222)	20.9
Digestive organs	61 (37–85)	8.9	−10 (−26–6)	4.6	101 (62–141)	11.8
Infections	35 (21–49)	5.1	25 (12–37)	11.5	86 (57–116)	10.1
Endocrine system	31 (17–45)	4.5	10 (0–20)	4.6	30 (10–50)	3.5
Benign and *in situ* neoplasms	18 (5–31)	2.6	25 (13–38)	11.5	2 (−12–15)	0.2
Nervous system and sense organs	18 (6–30)	2.6	10 (0–20)	4.6	25 (6–45)	2.9
Nonmalignant haematological conditions	12 (4–20)	1.8	10 (3–17)	4.6	39 (20–57)	4.6
Skin and subcutaneous tissue	12 (2–22)	1.8	6 (−4–15)	2.8	24 (5–43)	2.8
Urinary system and genital organs	10 (−9–29)	1.5	7 (−9–22)	3.2	53 (22–84)	6.2
Eye	8 (0–16)	1.2	2 (−3–7)	0.9	−1 (−7–5)	−0.1
Musculoskeletal and connective tissue	4 (−11–20)	0.6	−14 (−27–1)	−6.5	28 (1–55)	3.3
Ear and mastoid	−3 (−6–1)	−0.4	0 (−4–4)	0.0	2 (−6–9)	0.2
**Total**	**682 (614–751)**	**100.0**	**217 (168–265)**	**100.0**	**855 (751–960)**	**100.0**
**Special calculation for infections**						
Infections classified in other ICD–chapters^e^	58 (40–76)	8.5	40 (24–55)	18.4	146 (107–184)	17.1
All infections^f^	93 (70–116)	13.6	64 (44–84)	29.5	232 (183–280)	27.1

a No information on rehospitalisations available.

b No rehospitalisation with Hodgkin lymphoma as primary diagnosis in an oncological or haematological department one year or more after the first Hodgkin lymphoma diagnosis or, if rehospitalised for Hodgkin lymphoma 6 months to one year after the first Hodgkin lymphoma diagnosis, did not die within 10 years after the first Hodgkin lymphoma diagnosis.

c Rehospitalisation with Hodgkin lymphoma as primary diagnosis in an oncologic or haematologic department one year or more after the first Hodgkin lymphoma diagnosis, or rehospitalisation 6 months to one year after the first Hodgkin lymphoma diagnosis and deceased within 10 years of the first Hodgkin lymphoma diagnosis.

d Absolute excess rate per 10,000 person‐years; the rates are standardised for age, sex and calendar periods.

e From **Nervous system and sense organs**: bacterial meningitis, encephalitis, myelitis and encephalomyelitis and intracranial and intraspinal abscess; from **Circulatory system**: Acute and subacute endocarditis; from **Respiratory system**: Acute upper respiratory infections, pneumonia, other acute lower respiratory infections, bronchitis, abscess of lung and pleural empyema; from **Digestive organs**: acute peritonitis; from **Skin and subcutaneous tissue**: cutaneous abscess, furuncle and carbuncle, cellulitis, other infections of skin and subcutaneous tissue; from **Musculoskeletal and connective tissue**: infectious arthropathies, from **Urinary and genital organs**: cystitis.

f The sum of infections classified in the ICD chapter ‘Infections’ and infections classified in other ICD chapters.

Table 4 gives further details regarding the 145 specific disease categories and shows that the rate ratio for hospitalisation for all types of infections combined was significantly higher in the assumed relapse subcohort (RR, 5.3) than in the early mixed subcohort (RR, 2.5) or the primary treatment only subcohort (RR, 2.2). We did a separate analysis of the rate ratios of hospitalisation for infections prior to the Hodgkin lymphoma diagnosis. Interestingly, this analysis showed a modest increase in risk from birth to three years prior to the diagnosis of Hodgkin lymphoma (RR, 1.4, 95% CI, 1.1–1.8) and a more marked increase in the three‐year period preceding the diagnosis (2.4; 1.5–3.8). The latter finding likely reflects the consequences of a yet undiagnosed Hodgkin lymphoma on the immune system or disorders of the immune system or other diseases that could lead to Hodgkin lymphoma or that have a shared aetiology.

**Table 4:** The cohort of 1,768 five‐year survivors of Hodgkin lymphoma diagnosed in adolescence or young adulthood divided into three subcohorts

	**Survivors of Hodgkin lymphoma diagnosed 1943–1976**			**Survivors of Hodgkin lymphoma diagnosed 1977–2004**					
	**Early mixed subcohort** a			**Primary treatment only subcohort** b			**Assumed relapse subcohort** c		
	**No. of new diagnosesobs**.	**RR(95% CI)**	**AER** d **(95% CI)**	**No. of new diagnosesobs**.	**RR(95% CI)**	**AER** d **(95% CI)**	**No. of new diagnoses obs**.	**RR(95% CI)**	**AER** d **(95% CI)**
**Total**	**1,519**	**2.0 (1.9–2.1)**	**682 (614–751)**	**805**	**1.5 (1.4–1.6)**	**217 (168–265)**	**614**	**2.9 (2.6–3.1)**	**855 (751–960)**
**Infections**	**63**	**2.6 (2.0–3.3)**	**35 (21–49)**	**53**	**2.1 (1.6–2.8)**	**25 (12–37)**	**50**	**5.1 (3.9–6.7)**	**86 (57–116)**
• Sepsis	13	3.0 (1.7–5.2)	8 (1–14)	16	5.5 (3.3–9.0)	11 (5–18)	16	14.7 (9.0–24.1)	32 (15–49)
**Malignant neoplasms**	**197**	**3.2 (2.8–3.7)**	**122 (97–147)**	**75**	**2.5 (2.0–3.1)**	**39 (24–54)**	**51**	**4.7 (3.6–6.2)**	**86 (56–116)**
• Digestive organs	34	2.9 (2.1–4.1)	20 (10–31)	13	3.1 (1.8–5.3)	8 (1–14)	8	5.2 (2.6–10.4)	14 (2–26)
‐ Colorectal cancer	16	2.3 (1.4–3.8)	8 (1–15)	6	2.4 (1.1–5.4)	3 (−1–7)	5	5.6 (2.3–13.5)	9 (−1–18)
• Respiratory system and intrathoracic organs	31	3.3 (2.3–4.7)	19 (10–29)	9	2.8 (1.4–5.3)	5 (0–10)	13	11.5 (6.7–20.0)	26 (10–41)
‐ Cancer of lung, bronchus and trachea	28	3.3 (2.3–4.8)	17 (8–27)	8	2.8 (1.4–5.6)	4 (0–9)	13	13.4 (7.8–23.2)	26 (11–41)
• Mesothelioma and connective tissue	10	13.2 (7.0–24.8)	8 (3–14)	2	4.9 (1.2–19.9)	1 (−1–4)	2	13.1 (3.2–52.7)	4 (−2–10)
‐ Mesothelioma	5	21.7 (8.8–53.5)	4 (0–8)	0	–	–	0	–	–
• Breast	38	4.1 (3.0–5.6)	26 (15–38)	22	3.8 (2.5–5.9)	14 (6–22)	9	5.2 (2.7–9.9)	16 (3–28)
• Lymphatic and haematopoietic tissue	20	4.6 (2.9–7.1)	14 (6–22)	5	2.0 (0.8–4.9)	2 (−2–6)	4	4.2 (1.6–11.2)	7 (−2–15)
‐ Non‐Hodgkin lymphoma	10	5.6 (3.0–10.4)	7 (2–13)	2	1.8 (0.5–7.4)	1 (−2–3)	0	–	–
‐ Leukaemia	9	5.7 (3.0–11.1)	7 (1–12)	2	2.6 (0.7–10.6)	1 (−1–3)	3	10.7 (3.4–33.3)	6 (−1–13)
**Nonmalignant haematological conditions**	**22**	**2.6 (1.7**–**3.9)**	**12 (4**–**20)**	**17**	**3.1 (1.9**–**5.0)**	**10 (3**–**17)**	**20**	**9.7 (6.2**–**15.0)**	**39 (20**–**57)**
**Diseases of nervous system and sense organs**	**48**	**1.7 (1.3**–**2.3)**	**18 (6**–**30)**	**35**	**1.5 (1.1**–**2.1)**	**10 (0**–**20)**	**21**	**2.3 (1.5**–**3.5)**	**25 (6**–**45)**
• Bacterial meningitis	6	9.5 (4.2–21.5)	5 (1–9)	5	16.4 (6.7–40.3)	4 (0–8)	3	23.1 (7.4–72.8)	6 (−1–13)
**Circulatory system**	**458**	**2.7 (2.5**–**3.0)**	**259 (221**–**297)**	**167**	**1.8 (1.6**–**2.1)**	**66 (44**–**88)**	**129**	**3.6 (3.1**–**4.3)**	**201 (153**–**249)**
• Angina pectoris	46	2.6 (2.0–3.5)	26 (14–39)	18	1.8 (1.1–2.9)	7 (0–14)	16	4.3 (2.6–7.1)	27 (10–45)
• Acute myocardial infarction	53	2.9 (2.2–3.8)	32 (19–45)	14	1.7 (1.0–2.9)	5 (−1–12)	15	4.7 (2.8–7.7)	26 (9–42)
• Chronic ischemic heart disease	35	3.5 (2.5–4.9)	23 (12–33)	13	3.0 (1.7–5.2)	8 (1–14)	10	6.0 (3.2–11.1)	18 (5–32)
• Mitral valve disorders	12	11.5 (6.5–20.4)	10 (4–16)	4	9.9 (3.7–26.9)	3 (0–7)	1	6.4 (0.9–45.7)	2 (−2–6)
• Aortic valve disorders	44	24.7 (18.2–33.5)	38 (27–50)	14	18.4 (10.7–31.4)	12 (5–18)	10	34.9 (18.6–65.7)	21 (8–34)
• Heart failure	36	7.1 (5.1–9.9)	28 (17–39)	14	6.2 (3.6–10.5)	10 (4–17)	3	3.4 (1.1–10.7)	5 (−3–12)
**Respiratory system**	**171**	**2.6 (2.3**–**3.0)**	**95 (72**–**118)**	**98**	**2.0 (1.6**–**2.4)**	**42 (25**–**59)**	**103**	**5.3 (4.4**–**6.4)**	**179 (137**–**222)**
• Pneumonia	57	3.0 (2.3–3.9)	35 (21–49)	35	2.6 (1.9–3.7)	19 (9–29)	40	8.0 (5.8–10.9)	78 (50–105)
**Digestive organs**	**188**	**1.6 (1.4**–**1.8)**	**61 (37**–**85)**	**88**	**0.9 (0.7**–**1.1)**	–**10 (**–**26**–**6)**	**87**	**2.2 (1.8**–**2.7)**	**101 (62**–**141)**
**Urinary system and genital organs**	**118**	**1.1 (0.9**–**1.3)**	**10 (**–**9**–**29)**	**86**	**1.1 (0.9**–**1.4)**	**7 (**–**9**–**22)**	**54**	**1.8 (1.4**–**2.4)**	**53 (22**–**84)**
• Renal failure	9	4.0 (2.1–7.7)	6 (1–11)	3	1.9 (0.6–6.0)	1 (−2–4)	5	8.4 (3.5–20.2)	9 (0–19)
**Special calculations for infections**									
• Infections classified in other ICD‐chapters^e^	109	2.5 (2.0–3.0)	58 (40–76)	84	2.2 (1.8–2.7)	40 (24–55)	83	5.5 (4.4–6.8)	146 (107–184)
• All infections^f^	172	2.5 (2.2–2.9)	93 (70–116)	137	2.2 (1.8–2.6)	64 (44–84)	133	5.3 (4.5–6.3)	232 (183–280)

Rate ratios (RRs) and absolute excess rates (AERs) with corresponding 95% confidence intervals (CIs) are given for selected main diagnostic groups and for selected specific disease categories.

a No information on rehospitalisations available.

b No rehospitalisation with Hodgkin lymphoma as primary diagnosis in an oncological or haematological department one year or more after the first Hodgkin lymphoma diagnosis or, if rehospitalised for Hodgkin lymphoma 6 months to one year after the first Hodgkin lymphoma diagnosis, did not die within 10 years after the first Hodgkin lymphoma diagnosis.

c Rehospitalisation with Hodgkin lymphoma as primary diagnosis in an oncologic or haematologic department one year or more after the first Hodgkin lymphoma diagnosis, or rehospitalisation 6 months to one year after the first Hodgkin lymphoma diagnosis and deceased within 10 years of the first Hodgkin lymphoma diagnosis.

d Absolute excess rate per 10,000 person‐years; the rates are standardised for age, sex and calendar periods.

e From **Nervous system and sense organs**: bacterial meningitis, encephalitis, myelitis and encephalomyelitis and intracranial and intraspinal abscess; from **Circulatory system**: Acute and subacute endocarditis; from **Respiratory system**: Acute upper respiratory infections, pneumonia, other acute lower respiratory infections, bronchitis, abscess of lung and pleural empyema; from **Digestive organs**: acute peritonitis; from **Skin and subcutaneous tissue**: cutaneous abscess, furuncle and carbuncle, cellulitis, other infections of skin and subcutaneous tissue; from **Musculoskeletal and connective tissue**: infectious arthropathies, from **Urinary and genital organs**: cystitis.

f The sum of infections classified in the ICD chapter ‘Infections’ and infections classified in other ICD chapters.

Among the specific disease categories, the highest AERs were seen for aortic valve disorders, acute myocardial infarction, angina pectoris, heart failure, chronic ischaemic heart disease, pneumonia, cancers of the breast, respiratory system and digestive organs and sepsis (Table 4). The AERs for malignant neoplasms and heart diseases tended to be highest in the early mixed cohort, while the AERs for infections tended to be highest in the 1977–2004 assumed relapse subcohort. Results for all 145 specific diseases are shown in Supporting Information Table S2.

**Figure 3:**
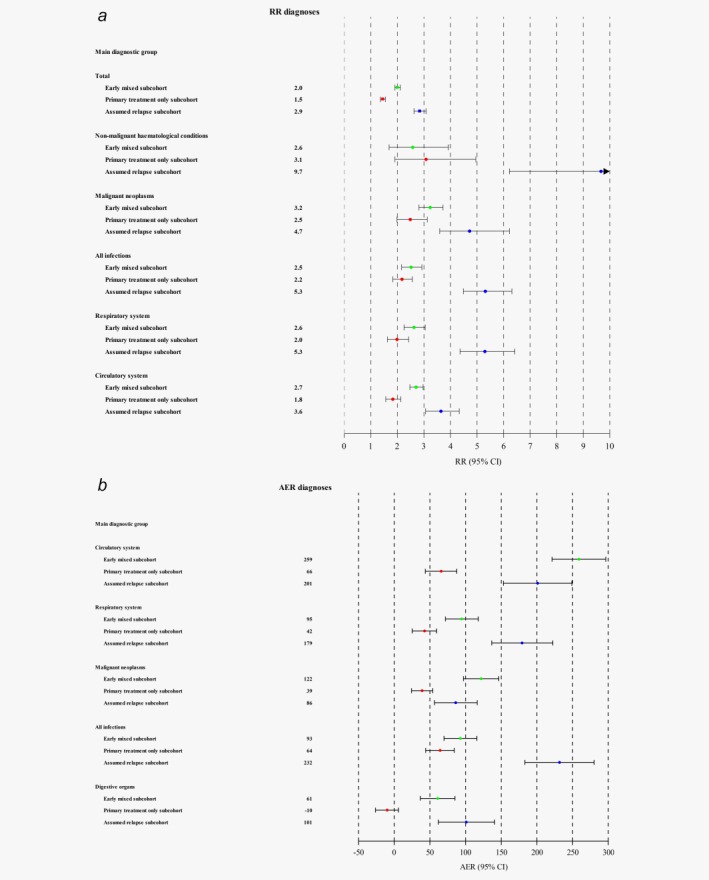
(*a*) Rate ratios (RRs) for bed‐days for selected main diagnostic groups with estimates for each of the three subcohorts. (*b*) Absolute excess rates (AERs) for bed‐days for selected main diagnostic groups per 10,000 person‐years for each of the three subcohorts. The total AERs (not included in the plot) are:‐ for those diagnosed 1943–1976 (i.e., early mixed subcohort): 20,520, 95% CI, 20,205–20,836; for those diagnosed 1977–2004 and with no rehospitalisations (i.e., primary treatment only subcohort): 3,384, 95% CI, 3,226–3,542; for those diagnosed 1977–2004 and with rehospitalisations (i.e., assumed relapse subcohort): 37,070, 95% CI, 36,489–37,652. [Color figure can be viewed at wileyonlinelibrary.com]

The largest RRs for bed‐days were for malignant neoplasms, nonmalignant haematological conditions, diseases of the respiratory system, diseases of the circulatory system and all infections; all with significant heterogeneity between the three subcohorts of Hodgkin lymphoma survivors. In general, the proportional increases in the bed‐day rates were highest for the 1977–2004 assumed relapse subcohort, followed by the early mixed subcohort and then the primary treatment only subcohort (Fig. 3, panel A). The largest AERs for bed‐days were for malignant neoplasms, circulatory diseases and all infections combined, with a pattern between the three subcohorts similar to that seen for the RRs (Fig. 3, panel B). The many extra bed‐days among survivors of Hodgkin lymphoma were mainly due to more frequent hospitalisations rather than hospitalisations over longer periods.

Among the 887 survivors diagnosed during 1977–2004 and with no readmission to hospital for Hodgkin lymphoma, 494 only received chemotherapy as judged from the records of the Cancer Registry while 338 survivors also received radiotherapy, and the overall rate ratios for hospitalisation in these two groups were 1.3 (95% CI,1.2–1.4) and 1.6 (1.4–1.7), respectively. Ratios also tended to be higher in the group who received both radiotherapy and chemotherapy for the main diagnostic groups of malignant neoplasms (RR, 3.0 vs. 2.0), nonmalignant haematological conditions (4.0 vs. 2.3), diseases of circulatory system (2.1 vs. 1.6) and diseases of respiratory system (2.5 vs. 1.5), although none of the differences was statistically significant.

## Discussion

In this population‐based long‐term follow‐up study of 1,768 five‐year survivors of Hodgkin lymphoma diagnosed in adolescence or young adulthood in Denmark, survivors received new diagnoses requiring hospitalisation significantly more often than the general population, and survivors spent more days in hospital. The increased burden of disease affected both men and women and was lifelong and particularly heavy for patients readmitted to hospital for Hodgkin lymphoma and who are likely to have had a relapse. Thus, for example, among patients aged 50–59 years diagnosed in 1977–2004 and readmitted to hospital for an assumed relapse 15 in 100 experienced a new disease requiring inpatient care each year. The equivalent estimate in the primary treatment only subcohort was 5 in 100. All main diagnostic groups were affected but diseases of the circulatory system, all infections combined, and malignant neoplasms explained together between 61% and 78% of the excess new diagnoses depending on the subcohort of survivors.

To our knowledge, the present study is the first to describe the long‐term risks of hospitalisation for the full spectrum of physical diseases in survivors of Hodgkin lymphoma diagnosed in adolescence or young adulthood in a large population. One other study has done so^18^ but, as it was based on only 281 individuals, it was unable to provide risk estimates with any precision. Several studies of cause‐specific mortality in survivors of Hodgkin lymphoma diagnosed in childhood or early adult life have been carried out,^19^, ^20^, ^21^ and the risk of hospitalisation among survivors of all types of cancer diagnosed in adolescence and young adulthood has been studied in other populations.^22^, ^23^, ^24^, ^25^ Other studies specifically of survivors of Hodgkin lymphoma diagnosed in young adulthood have usually reported on specific risks of second cancers^11^, ^26^, ^27^, ^28^, ^29^, ^30^, ^31^, ^32^, ^33^ or cardiovascular diseases.^12^, ^34^, ^35^, ^36^ The results of our study are in agreement with their findings. In particular, we found substantial proportional increases in the incidence of aortic valve disease and of female breast cancer. Our study is, however, the first large study that has been able to quantify the burden of different diseases and compare them. We have shown that, while cardiovascular disease is responsible for by far the largest number of new diagnoses, by far the largest number of days spent in hospital are attributable to cancer. Our study has also, for the first time, revealed the importance of infections as a late effect of Hodgkin lymphoma, both in terms of numbers of new diagnoses and in terms of time spent in hospital (Figs. 2 and 3). Several other studies have reported increases in incidence or mortality from infections either in survivors of Hodgkin lymphoma,^9^, ^18^ or in broader groups of cancers diagnosed in teenagers and young adults^22^, ^24^, ^25^ but none of these studies has revealed the extent of the disease burden from infections, as they have all considered only infections classified in Chapter II of the ICD, rather than bringing together all infections irrespective of where they appear in the ICD.

We included patients treated over a time period of 60 years during which time the treatment for Hodgkin lymphoma has evolved dramatically. The treatments used in Denmark during this time period are similar to those used in other developed countries. In the period 1943–1964 radiotherapy was the mainstay of treatment for Hodgkin lymphoma; initially given as palliative treatment, although later as intended curative treatment in the form of total or subtotal nodal radiotherapy.^37^ In the period 1965–1980 radiotherapy was still the primary treatment but now with adjuvant chemotherapy. Around 1980 chemotherapy became the primary treatment, with radiotherapy as consolidating treatment. From approximately 1970, the mustargen–oncovin–procarbazine–prednisone (MOPP) regime was used; however, a gradual shift to an adriamycin–bleomycin–vinblastine–dacarbazine (ABVD) regime took place around 1985. Without systemic treatment, the risk of relapse and the need for further treatment was much higher than in the more recent period, when combination treatment with chemotherapy became the standard. Thus the subsequent morbidity of many patients previously treated for Hodgkin lymphoma with radiotherapy alone was heavily influenced by intensive chemotherapy and perhaps further radiotherapy given for relapse. As this study lacks individual and detailed treatment data, we cannot draw any conclusions regarding the specific links between types of treatment and risks of physical disease. However, for the subcohort of survivors diagnosed during 1977–2004, we saw a tendency for higher risks among those registered in the Danish Cancer Registry as treated with radiotherapy compared to those registered without.

The prospective nature of this study, with virtually complete registration of cases of Hodgkin lymphoma in adolescence and young adulthood prior to, and independently of, the recording of subsequent hospital admissions, eliminates the possibility of selection bias and differential reporting. However, our study also has some important limitations. Conditions such as less severe late effects that are treated exclusively in outpatient clinics or in the primary healthcare system, will not have been included, implying that the total burden of physical disease experienced by Hodgkin lymphoma survivors may be somewhat underestimated. On the other hand, the diseases included in our study represent the most severe segment of late effects, which are also the most important ones to consider when planning programmes for surveillance and intervention. We cannot exclude the possibility that surveillance bias affects our study, since the health professional's knowledge about their patient being a survivor of Hodgkin lymphoma might influence their decisions on whether to hospitalise. This might have led to an unknown degree of overestimation of the reported risk estimates.

We applied a large set of statistical tests on the material, which implies that on average one out of 20 findings may be false positive. However, our survey should not be regarded as a study testing a specific hypothesis but rather a study describing a wide range of potential late effects in five‐year survivors of Hodgkin lymphoma.

Follow‐up programmes have also changed dramatically over the period. Since 2000, survivors of Hodgkin lymphoma in Denmark are followed approximately every fourth month during the first two years after the end of treatment and thereafter approximately twice a year until five years after the end of treatment. Before 2000, the norm was follow‐up visits twice a year 10 years after the end of treatment; at these visits scans were performed to detect any abnormalities; nowadays scans are primarily performed when the survivor reports symptoms which could indicate abnormalities or give suspicions about relapse.

Survivors of Hodgkin lymphoma diagnosed in adolescence or young adulthood are at increased risk for a wide range of physical diseases severe enough to require hospitalisation. This study is the first to quantify the burden of the full spectrum of physical diseases suffered by this population. However, research is needed to confirm the assumption that screening, surveillance and intervention among Hodgkin lymphoma survivors can be effective in detecting and modifying the course of diseases that occur as a result of their previous diagnosis and treatment. If the efficacy of such programs can be demonstrated and they can be implemented in a cost‐effective manner, then the considerable burden of disease quantified in this study could be reduced and the potential to improve both the quality and length of life for Hodgkin lymphoma survivors would be substantial.

## Supporting information

Supporting Information Figure

Supporting Information Table 1

Supporting Information Table 2
