# Supplementary material for: Long‐term hospitalisation rates among 5‐year survivors of Hodgkin lymphoma in adolescence or young adulthood: A nationwide cohort study
Source: Int J Cancer. 2017 Mar 14;140(10):2232–45. doi: 10.1002/ijc.30655 (PMC5396317; doi:10.1002/ijc.30655)
Supplement: Supplementary file 1 — Supporting Information Figure [file IJC-140-2232-s001.docx]

Figure S1. Rates per 10,000 person-years of new diagnoses requiring hospitalisation for 494 Hodgkin lymphoma survivors diagnosed during 1943-1976 (ie early mixed subcohort) (panel A), for 887 survivors with no re-hospitalisations (ie primary treatment only subcohort) (panel B) and for 387 survivors with re-hospitalisations (ie assumed relapse subcohort) (panel C). For the same three groups of survivors rates of bed-days are shown in panel D-F. Rates are standardized for gender and calendar period.
