# Supplementary material for: Long‐term hospitalisation rates among 5‐year survivors of Hodgkin lymphoma in adolescence or young adulthood: A nationwide cohort study
Source: Int J Cancer. 2017 Mar 14;140(10):2232–45. doi: 10.1002/ijc.30655 (PMC5396317; doi:10.1002/ijc.30655)
Supplement: Supplementary file 2 — Supporting Information Table 1 [file IJC-140-2232-s002.docx]

| **Table S1. Equivalent grouping of codes of the International Classification of Diseases, eighth revision (ICD-8) and tenth revision (ICD-10) used for analysis of the disease burden among 1768 five-year survivors of adolescent and young adult Hodgkin lymphoma** | | | |
| --- | --- | --- | --- |
| **Main diagnostic group (14 groups)** | | **ICD-8** | **ICD-10** |
| Specific disease category (145 categories) | |  |  |
| **Infections** | |  | **A00-B99** |
| Intestinal infectious diseases | | 000-009 | A00-A09 |
| Tuberculosis | | 010-019 | A15-A19 |
| Sepsis | | 038 | A40-A41 |
| Other bacterial diseases | | 020-027, 030-037, 039 | A20-A28, A43-A49 |
| Syphilis and other venereal diseases | | 090-104 | A50-A74 |
| Herpes zoster | | 053 | B02 |
| Viral hepatitis | | 070 | B15-B19 |
| HIV disease | | - | B20-B24 |
| Other viral diseases | | 040-046, 050-057, 060-079 | A80-A89, A92-A99, B00-B09, B25-B34 |
| Candidiasis | | 112 | B37 |
| Other mycoses | | 110-111, 113-117 | A42, B35-B36, B38-B49 |
| Other infections | | 080-089, 120-129, 130-136 | A75-A79, B50-B99 |
| **Malignant neoplasms^a^** | |  |  |
| Cancer of buccal cavity and pharynx | |  | C00-C14, C46.2 |
| Cancer of digestive organs | |  | C15-C26 |
| Cancer of respiratory system and intrathoracic organs | |  | C30-C39 |
| Cancer of bones, joints and articular cartilage | |  | C40-C41 |
| Malignant melanoma of skin | |  | C43 |
| Mesothelioma and connective tissue | |  | C45.0-C45.9, C46.1, C46.3, C46.7, C46.8, C46.9, C47-C49, B210 |
| Cancer of breast | |  | C50 |
| Cancer of female genital organs | |  | C51-C58 |
| Cancer of male genital organs | |  | C60-C63 |
| Cancer of urinary tract | |  | C64-C68, D09.0-D09.1, D30.1-D30.9, D41.1-D41.9 |
| Cancers of eye, brain and other parts of CNS | |  | C69-C72, C75.1-C75.3, D32-D33, D35.2-D35.4, D42-D43, D44.3-D44.5 |
| Cancer of endocrine organs | |  | C73-C74, C75.0, C75.4-C75.9 |
| Cancer of lymphatic and haematopoietic tissue | |  | C81-C96 |
| Cancer of ill-defined and unspecified sites | |  | C76-C80 |
|  | |  |  |
| **Table S1. Continued.** | | | |
| **Main diagnostic group** | **ICD-8** | | **ICD-10** |
| Disease category |  | |  |
| **Benign and in situ neoplasms** | 210-223.0, 224, 225.5-225.6, 226.0-226.1, 226.4-228 | | D00-D05, D07-D30.0, D31, D34-D35.1, D35.5-D36 |
| **Non-malignant haematological** |  | | **D50-D89** |
| Anaemias | 280-285 | | D50-D64 |
| Coagulation defects, purpura and other haemorrhagic conditions | 286-287 | | D65-D69 |
| Other diseases of blood and blood-forming organs | 288-289 | | D70-D89 |
| **Endocrine** |  | | **E00-E90** |
| Non-toxic goitre | 240-241 | | E04 |
| Other disorders of thyroid gland | 242-246 | | E00-E03 |
| Diabetes mellitus and related conditions | 250-251 | | E10-E16 |
| Cushing syndrome | 255 | | E24 |
| Ovarian dysfunction | 256 | | E28 |
| Diseases of other endocrine organs | 252-254, 257-258 | | E20-E23, E25-E27, E29-E35 |
| Dehydration and disorders of fluid and electrolyte balance | - | | E86-E87 |
| Other metabolic and nutritional disturbances | 260-279 | | E40-E68, E70-E83, E88-E90 |
| **Nervous system and sense organs** |  | | **G00-G99** |
| Bacterial meningitis | 320 | | G00 |
| Encephalitis, myelitis and encephalomyelitis | 323 | | G04 |
| Intracraniel and intraspinal abscess | 322 | | G06 |
| Epilepsy | 345 | | G40-G41 |
| Migraine | 346 | | G43-G44 |
| Transient cerebral ischaemic attacks | 435 | | G45 |
| Nerve, nerve root and plexus disorders | 350-353, 356-358 | | G50-G59 |
| Polyneuropathies | 354-355 | | G60-G63 |
| Para-, hemi- and tetraplegia | - | | G81-G83 |
| Other diseases of nervous system | 321, 324, 330-333, 340-344, 347-349 | | G01-G02, G05, G07-G09, G20-G26, G30-G32, G35-G37, G46-G47, G64, G90-G99 |
| **Eye** |  | | **H00-H59** |
| Inflammatory diseases of the eye | 360-369 | | H00-H22, H30 |
| Other diseases and conditions of the eye | 370-373, 375-379 | | H31-H36, H43-H59 |
| Cataract | 374 | | H25-H28, H40-H42 |
| **Ear and mastoid** |  | | **H60-H95** |
| Diseases of middle ear and mastoid process | 381-383 | | H65-H75 |
| Diseases of inner ear | 385-386 | | H80-H83 |
| Other diseases of ear | 380, 384, 387-389 | | H60-H62, H90-H95 |
| **Table S1. Continued.** | | | |
| **Main diagnostic group** | **ICD-8** | | **ICD-10** |
| Disease category |  | |  |
| **Circulatory system** |  | | **I00-I99** |
| Hypertensive disease | 400-404 | | I10-I15 |
| Angina pectoris | 413 | | I20 |
| Acute myocardial infarction | 410-411 | | I21-I24 |
| Chronic ischaemic heart disease | 412 | | I25 |
| Pulmonary embolism | 450 | | I26 |
| Acute and chronic diseases of pericardium | 420, 423 | | I30-I32 |
| Acute and sub-acute endocarditis | 421 | | I33, I38-I39 |
| Mitral valve disorders | 394, 396, 424 | | I34 |
| Aortic valve disorders | 395 | | I35 |
| Cardiomyopathy | 425-426 | | I42-I43 |
| Atrioventricular block and other conduction disorders | 427 | | I44-I45 |
| Cardiac arrest | - | | I46 |
| Paroxysmal tachycardia | - | | I47 |
| Atrial fibrillation and flutter | - | | I48 |
| Other cardiac arrhythmias | - | | I49 |
| Heart failure | 428 | | I50 |
| Other heart diseases | 414, 422, 429 | | I00-I09, I27-I28, I36-I37, I40-I41, I51-I52 |
| Intracranial haemorrhage | 430-431 | | I60-I62 |
| Cerebral infarction | 433-434 | | I63 |
| Stroke NOS^b^ | 436 | | I64 |
| Other cerebrovascular diseases | 432, 437-438 | | I65-I69 |
| Atriosclerosis | 440 | | I70 |
| Atrial embolism and thrombosis | 444 | | I74 |
| Other peripheral vascular diseases | 441-443, 445-448 | | I71-I73, I77-I79 |
| Phlebitis and thrombophlebitis | 451 | | I80 |
| Venous embolism and thrombosis | 452 | | I81-I82 |
| Other disorders of circulatory system | 454-458 | | I83-I89, I95-I99 |
| **Respiratory** |  | | **J00-J99** |
| Acute upper respiratory infections | 460-465 | | J00-J06 |
| Diseases of vocal cords and larynx | 506 | | J38 |
| Other diseases of upper respiratory tract | 500-505, 507-508 | | J30-J37, J39 |
| Pneumonia | 480-486 | | J12-J18 |
| Other acute lower respiratory infections | 466 | | J20-J22 |
| Bronchitis | 490-491 | | J40-J42 |
| Chronic obstructive pulmonary disease | 492, 518 | | J43-J44 |
| Asthma | 493 | | J45-J46 |
| Lung diseases due to external agents | 515-516 | | J60-J70 |
| Pulmonary oedema and other interstitial  diseases | 514, 517 | | J80-J84 |
| **Table S1. Continued.** | | | |
| **Main diagnostic group** | **ICD-8** | | **ICD-10** |
| Disease category |  | |  |
| Abscess of lung and pleural empyema | 510, 513 | | J85-J86 |
| Pneumothorax | 512 | | J93 |
| Pleural effusion and other pleural conditions | 511 | | J90-J92, J94 |
| Respiratory failure | - | | J96 |
| Other respiratory disorders | 470-474, 519 | | J09-J11, J46, J95, J98-J99 |
| **Digestive** |  | | **K00-K93** |
| Diseases of oral cavity, salivary glands and jaws | 520-529 | | K00-K14 |
| Oesophagitis and other diseases of  oesophagus | 530 | | K20-K23 |
| Peptic ulcer of stomach and duodenum | 534-537 | | K25-K27 |
| Other diseases of stomach and duodenum | 534-537 | | K28-K31 |
| Hernia | 544-553 | | K40-K46 |
| Crohn disease and ulcerative colitis | 563 | | K50-K51 |
| Other non-infective gastroenteritis and colitis | 561 | | K52 |
| Paralytic ileus without hernia | 560 | | K56 |
| Other functional intestinal disorders | 564, 568 | | K57-K59 |
| Fissure, fistula and abscess of anal and rectal regions | 565-566 | | K60-K62 |
| Other diseases of intestine | 540-543, 562 | | K35-K38, K55, K63-K64 |
| Acute peritonitis | 567 | | K65 |
| Hepatic failure | 570-571 | | K72 |
| Other diseases of liver | 572-573 | | K70-K71, K73-K77 |
| Cholelithiasis | 574 | | K80 |
| Acute pancreatitis | 577 | | K85 |
| Other disease of digestive system | 569, 575-576 | | K66-K67, K81-K84, K86-K87, K90-K93 |
| **Skin and subcutaneous tissue** |  | | **L00-L99** |
| Cutaneous abscess, furuncle and carbuncle | 680, 685 | | L02 |
| Cellulitis | 681 | | L03 |
| Pyoderma and erythrasma | 682 | | L08 |
| Other infections of skin and subcutaneous tissue | 683-684, 686 | | L00-L01, L04-L05 |
| Other diseases of skin and subcutaneous tissue | 690-698, 700-709 | | L10-L99 |
| **Musculoskeletal and connective tissue** |  | | **M00-M99** |
| Infectious arthropathies | 710 | | M00-M03 |
| Rheumatoid arthritis | 711-712 | | M05-M06 |
| Arthrosis | 713-715 | | M20-M25 |
| Systemic connective tissue disorders | 716-717 | | M30-M36 |
|  |  | |  |
| **Table S1. Continued.** | | | |
| **Main diagnostic group** | **ICD-8** | | **ICD-10** |
| Disease category |  | |  |
| Kyphosis, lordosis and other deforming dorsopathies | 721 | | M40-M43 |
| Spondylopathies | 720 | | M45-M49 |
| Intervertebral disc disorders | 725-726 | | M50-M51 |
| Other dorsopathies | 728 | | M52-M54 |
| Disorders of muscles | 732-733 | | M60-M63 |
| Other soft tissue disorders | 730-731, 734 | | M65-M79 |
| Osteopathies and chondropathies | 722 | | M80-M94 |
| Other disorders of musculoskeletal system and connective tissue | 723-724, 727-729 | | M07-M14, M95-M99 |
| **Urinary system and genital** |  | | **N00-N99** |
| Glomerular diseases | 580, 582-583 | | N00-N08 |
| Tubulointestinal nephritis | 581-590 | | N10-N12 |
| Obstructive and reflux uropathy | 591 | | N13 |
| Renal failure | 584 | | N17-N19 |
| Ureterolithiasis | 592 | | N20-N23 |
| Other disorders of kidney | 593 | | N14-N16, N25-N29 |
| Cystitis | 595 | | N30 |
| Urethral stricture | 598 | | N35 |
| Other disorders of urinary system | 594, 596-597, 599 | | N32-N34, N36-N39 |
| Hyperplasia of prostate | 600 | | N40 |
| Other diseases of male genital organs | 601-607 | | N41-N51 |
| Disorders of breast | 610-611 | | N60-N64 |
| Inflammatory diseases of female genital  organs | 612-615, 620, 622 | | N70-N77 |
| Endometriosis | 616 | | N80 |
| Other non-inflammatory disorders of female genital organs | 621, 623-629 | | N81-N98 |
| Other disorders of genitourinary system | - | | N99 |

^a^A conversion system was developed by the Danish Cancer Registry to translate the International Classification of Diseases for Oncology (ICD-O) and ICD-7 codes into ICD-10 codes. ICD-10 is the coding system used presently by the Danish Cancer Registry

^b^NOS = not otherwise specified

Note that we decided to include neoplasms of the urinary organs that were benign or of uncertain nature, and carcinomas in situ of the urinary tract in the category of ‘cancer of the urinary tract’ as these tumours often have an aggressive course. Non-melanoma skin cancer (ICD code C44) was not included since this disease is primarily treated in the primary health care system and does not require hospitalisation.
