# Supplementary material for: Long‐term hospitalisation rates among 5‐year survivors of Hodgkin lymphoma in adolescence or young adulthood: A nationwide cohort study
Source: Int J Cancer. 2017 Mar 14;140(10):2232–45. doi: 10.1002/ijc.30655 (PMC5396317; doi:10.1002/ijc.30655)
Supplement: Supplementary file 3 — Supporting Information Table 2 [file IJC-140-2232-s003.docx]

| Table S2. The cohort of 1768 five-year survivors of Hodgkin lymphoma diagnosed in adolescence and young adulthood divided into three subcohorts. Rate ratios (RRs) and absolute excess rates (AERs) with corresponding 95% confidence intervals (CIs) are given for each of the 14 main diagnostic groups and for each of the 145 specific disease categories. | | | | | | | | | | | |
| --- | --- | --- | --- | --- | --- | --- | --- | --- | --- | --- | --- |
|  | **Survivors of Hodgkin lymphoma diagnosed**  **1943-1976** | | |  | **Survivors of Hodgkin lymphoma diagnosed**  **1977-2004** | | | | | | |
|  | **Early mixed subcohort^a^** | | |  | **Primary treatment only subcohort^b^** | | |  | **Assumed relapse subcohort^c^** | | |
|  | **No. of new diagnoses**  **obs.** | **RR**  **(95% CI)** | **AER^d^**  **(95% CI)** |  | **No. of new diagnoses**  **obs.** | **RR**  **(95% CI)** | **AER^d^**  **(95% CI)** |  | **No. of new diagnoses obs.** | **RR**  **(95% CI)** | **AER^d^**  **(95% CI)** |
| **Infections** | **63** | **2.6 (2.0-3.3)** | **35 (21-49)** |  | **53** | **2.1 (1.6-2.8)** | **25 (12-37)** |  | **50** | **5.1 (3.9-6.7)** | **86 (57-116)** |
| Intestinal infectious diseases | 12 | 2.1 (1.2-3.7) | 6 (0-12) |  | 8 | 1.0 (0.5-2.1) | 0 (-5-5) |  | 4 | 1.3 (0.5-3.4) | 2 (-7-10) |
| Tuberculosis | 1 | 1.5 (0.2-10.8) | 0 (-1-2) |  | 1 | 2.2 (0.3-15.7) | 0 (-1-2) |  | 0 | - | - |
| Sepsis | 13 | 3.0 (1.7-5.2) | 8 (1-14) |  | 16 | 5.5 (3.3-9.0) | 11 (5-18) |  | 16 | 14.7 (9.0-24.1) | 32 (15-49) |
| Other bacterial diseases | 9 | 1.9 (1.0-3.6) | 4 (-2-9) |  | 6 | 1.3 (0.6-2.9) | 1 (-3-5) |  | 2 | 1.1 (0.3-4.4) | 0 (-6-6) |
| Syphilis and other venereal diseases | 4 | 2.5 (0.9-6.7) | 2 (-1-6) |  | 5 | 2.3 (1.0-5.6) | 3 (-1-6) |  | 5 | 5.8 (2.4-13.9) | 9 (-1-18) |
| Herpes zoster | 6 | 12.1 (5.3-27.2) | 5 (1-9) |  | 2 | 10.7 (2.6-43.8) | 2 (-1-4) |  | 4 | 58.9 (21.4-161.9) | 8 (0-17) |
| Viral hepatitis | 1 | 1.1 (0.2-8.0) | 0 (-2-2) |  | 2 | 1.7 (0.4-6.7) | 1 (-2-3) |  | 2 | 3.9 (1.0-15.7) | 3 (-3-9) |
| HIV disease | 0 | - | - |  | 1 | 1.7 (0.2-12.0) | 0 (-1-2) |  | 0 | - | - |
| Other viral diseases | 11 | 3.1 (1.7-5.7) | 7 (1-13) |  | 10 | 2.9 (1.6-5.5) | 6 (0-11) |  | 8 | 5.8 (2.9-11.7) | 14 (2-26) |
| Candidiasis | 2 | 10.0 (2.5-40.7) | 2 (-1-4) |  | 0 | - | - |  | 5 | 82.2 (32.9-205.5) | 11 (1-20) |
| Other mycoses | 0 | - | - |  | 0 | - | - |  | 1 | 19.2 (2.7-139.6) | 2 (-2-6) |
| Other infections | 4 | 2.6 (1.0-6.8) | 2 (-1-6) |  | 2 | 2.2 (0.5-8.7) | 1 (-1-3) |  | 3 | 7.8 (2.5-24.2) | 6 (-2-13) |
| **Malignant neoplasms** | **197** | **3.2 (2.8-3.7)** | **122 (97-147)** |  | **75** | **2.5 (2.0-3.1)** | **39 (24-54)** |  | **51** | **4.7 (3.6-6.2)** | **86 (56-116)** |
| Buccal cavity and pharynx | 5 | 2.7 (1.1-6.6) | 3 (-1-7) |  | 8 | 7.2 (3.6-14.5) | 6 (1-11) |  | 2 | 4.5 (1.1-18.0) | 3 (-3-9) |
| Digestive organs | 34 | 2.9 (2.1-4.1) | 20 (10-31) |  | 13 | 3.1 (1.8-5.3) | 8 (1-14) |  | 8 | 5.2 (2.6-10.4) | 14 (2-26) |
| Respiratory system and intrathoracic organs | 31 | 3.3 (2.3-4.7) | 19 (10-29) |  | 9 | 2.8 (1.4-5.3) | 5 (0-10) |  | 13 | 11.5 (6.7-20.0) | 26 (10-41) |
| Bones, joints and articular cartilage | 0 | - | - |  | 0 | - | - |  | 1 | 40.2 (5.4-298.3) | 2 (-2-6) |
| Malignant melanoma of skin | 4 | 1.5 (0.5-3.9) | 1 (-2-5) |  | 1 | 0.4 (0.1-2.8) | -1 (-3-0) |  | 1 | 1.1 (0.1-7.6) | 0 (-4-4) |
| Mesothelioma and connective tissue | 10 | 13.2 (7.0-24.8) | 8 (3-14) |  | 2 | 4.9 (1.2-19.9) | 1 (-1-4) |  | 2 | 13.1 (3.2-52.7) | 4 (-2-10) |
| Breast | 38 | 4.1 (3.0-5.6) | 26 (15-38) |  | 22 | 3.8 (2.5-5.9) | 14 (6-22) |  | 9 | 5.2 (2.7-9.9) | 16 (3-28) |
| Female genital organs | 11 | 2.4 (1.3-4.3) | 6 (0-12) |  | 4 | 1.8 (0.7-4.9) | 2 (-2-5) |  | 2 | 2.9 (0.7-11.5) | 3 (-3-9) |
| Male genital organs | 3 | 0.6 (0.2-1.7) | -2 (-5-1) |  | 1 | 0.4 (0.1-2.5) | -2 (-3-0) |  | 3 | 2.7 (0.9-8.3) | 4 (-3-11) |
| Urinary tract | 15 | 3.0 (1.8-5.1) | 9 (2-16) |  | 3 | 1.6 (0.5-4.9) | 1 (-2-4) |  | 3 | 4.2 (1.3-13.0) | 5 (-2-12) |
| Eye, brain and other parts of CNS | 10 | 3.4 (1.8-6.4) | 6 (1-12) |  | 4 | 1.8 (0.7-4.8) | 2 (-2-5) |  | 2 | 2.4 (0.6-9.6) | 3 (-3-8) |
| Endocrine organs | 5 | 19.1 (7.8-46.8) | 4 (0-8) |  | 0 | - | - |  | 1 | 7.0 (1.0-50.1) | 2 (-2-6) |
| Lymphatic and haematopoietic tissue | 20 | 4.6 (2.9-7.1) | 14 (6-22) |  | 5 | 2.0 (0.8-4.9) | 2 (-2-6) |  | 4 | 4.2 (1.6-11.2) | 7 (-2-15) |
| Ill-defined and unspecified sites | 11 | 6.0 (3.3-10.9) | 8 (2-14) |  | 3 | 4.2 (1.3-13.1) | 2 (-1-5) |  | 0 | - | - |
| **Benign and in situ neoplasms** | **54** | **1.6 (1.2-2.1)** | **18 (5-31)** |  | **55** | **2.1 (1.6-2.8)** | **25 (13-38)** |  | **10** | **1.1 (0.6-2.0)** | **2 (-12-15)** |
| **Non-malignant haematological** | **22** | **2.6 (1.7-3.9)** | **12 (4-20)** |  | **17** | **3.1 (1.9-5.0)** | **10 (3-17)** |  | **20** | **9.7 (6.2-15.0)** | **39 (20-57)** |
| Anaemias | 16 | 2.5 (1.5-4.1) | 9 (2-16) |  | 8 | 2.2 (1.1-4.4) | 4 (-1-9) |  | 13 | 9.9 (5.7-17.1) | 25 (10-40) |
| Coagulation defects, purpura and other haemorrhagic conditions | 4 | 4.6 (1.7-12.3) | 3 (-1-6) |  | 7 | 11.5 (5.4-24.4) | 6 (1-10) |  | 3 | 12.1 (3.9-37.8) | 6 (-1-13) |

| Table S2. Continued |  | | |  |  | | | | | | |
| --- | --- | --- | --- | --- | --- | --- | --- | --- | --- | --- | --- |
|  | **Survivors of Hodgkin lymphoma diagnosed**  **1943-1976** | | |  | **Survivors of Hodgkin lymphoma diagnosed**  **1977-2004** | | | | | | |
|  | **Early mixed subcohort^a^** | | |  | **Primary treatment only subcohort^b^** | | |  | **Assumed relapse subcohort^c^** | | |
|  | **No. of new diagnoses**  **obs.** | **RR**  **(95% CI)** | **AER^d^**  **(95% CI)** |  | **No. of new diagnoses**  **obs.** | **RR**  **(95% CI)** | **AER^d^**  **(95% CI)** |  | **No. of new diagnoses obs.** | **RR**  **(95% CI)** | **AER^d^**  **(95% CI)** |
| Other diseases of blood and blood-forming organs | 2 | 1.7 (0.4-6.7) | 1 (-2-3) |  | 2 | 1.6 (0.4-6.4) | 1 (-2-3) |  | 4 | 8.0 (3.0-21.4) | 8 (-1-16) |
| **Endocrine** | **62** | **2.3 (1.8-2.9)** | **31 (17-45)** |  | **33** | **1.5 (1.1-2.1)** | **10 (0-20)** |  | **22** | **2.7 (1.8-4.1)** | **30 (10-50)** |
| Non-toxic goitre | 18 | 4.1 (2.6-6.5) | 12 (5-20) |  | 15 | 4.1 (2.5-6.8) | 10 (3-17) |  | 2 | 1.5 (0.4-5.9) | 1 (-5-7) |
| Other disorders of thyroid gland | 7 | 2.8 (1.3-5.8) | 4 (-1-9) |  | 2 | 3.1 (0.8-12.4) | 1 (-1-4) |  | 2 | 7.8 (1.9-31.4) | 4 (-2-10) |
| Diabetes mellitus and related conditions | 16 | 1.5 (0.9-2.5) | 5 (-2-12) |  | 6 | 0.8 (0.4-1.8) | -1 (-5-3) |  | 8 | 2.6 (1.3-5.3) | 11 (-1-23) |
| Cushing syndrome | 0 | - | - |  | 1 | 8.4 (1.2-61.0) | 1 (-1-2) |  | 0 | - | - |
| Ovarian dysfunction | 0 | - | - |  | 0 | - | - |  | 0 | - | - |
| Diseases of other endocrine organs | 3 | 2.2 (0.7-6.9) | 1 (-2-5) |  | 0 | - | - |  | 0 | - | - |
| Dehydration and disorders of fluid and electrolyte balance | 15 | 3.9 (2.4-6.5) | 10 (3-17) |  | 1 | 0.4 (0.1-2.9) | -1 (-3-0) |  | 4 | 4.7 (1.8-12.6) | 7 (-2-15) |
| Other metabolic and nutritional disturbances | 3 | 0.8 (0.2-2.3) | -1 (-4-2) |  | 8 | 1.3 (0.7-2.7) | 2 (-3-7) |  | 6 | 2.9 (1.3-6.4) | 8 (-2-19) |
| **Diseases of nervous system and sense organs** | **48** | **1.7 (1.3-2.3)** | **18 (6-30)** |  | **35** | **1.5 (1.1-2.1)** | **10 (0-20)** |  | **21** | **2.3 (1.5-3.5)** | **25 (6-45)** |
| Bacterial meningitis | 6 | 9.5 (4.2-21.5) | 5 (1-9) |  | 5 | 16.4 (6.7-40.3) | 4 (0-8) |  | 3 | 23.1 (7.4-72.8) | 6 (-1-13) |
| Encephalitis, myelitis and encephalomyelitis | 1 | 3.9 (0.5-27.8) | 1 (-1-2) |  | 1 | 6.1 (0.8-43.7) | 1 (-1-2) |  | 2 | 30.1 (7.3-123.1) | 4 (-2-10) |
| Intracranial and intraspinal abscess | 0 | - | - |  | 1 | 6.7 (0.9-48.6) | 1 (-1-2) |  | 0 | - | - |
| Epilepsy | 7 | 1.4 (0.7-2.9) | 2 (-3-6) |  | 6 | 1.4 (0.6-3.0) | 1 (-3-6) |  | 2 | 1.1 (0.3-4.4) | 0 (-6-6) |
| Migraine | 4 | 1.2 (0.5-3.3) | 1 (-3-4) |  | 4 | 1.0 (0.4-2.8) | 0 (-3-4) |  | 0 | - | - |
| Transient cerebral ischaemic attacks | 11 | 1.9 (1.1-3.5) | 5 (-1-11) |  | 6 | 1.9 (0.8-4.1) | 2 (-2-7) |  | 3 | 2.5 (0.8-7.8) | 4 (-3-11) |
| Nerve, nerve root and plexus disorders | 4 | 0.7 (0.3-2.0) | -1 (-5-2) |  | 4 | 1.0 (0.4-2.7) | 0 (-3-3) |  | 3 | 1.9 (0.6-6.0) | 3 (-4-10) |
| Polyneuropathies | 1 | 0.9 (0.1-6.5) | 0 (-2-2) |  | 1 | 1.2 (0.2-8.6) | 0 (-2-2) |  | 2 | 5.9 (1.5-23.9) | 4 (-2-10) |
| Para-, hemi- and tetraplegia | 2 | 4.8 (1.2-19.2) | 1 (-1-4) |  | 2 | 2.5 (0.6-10.1) | 1 (-1-3) |  | 2 | 6.4 (1.6-25.8) | 4 (-2-10) |
| Other diseases of nervous system | 12 | 2.0 (1.1-3.5) | 5 (-1-11) |  | 5 | 0.9 (0.4-2.1) | -1 (-4-3) |  | 4 | 1.8 (0.7-4.7) | 4 (-5-12) |
| **Eye** | **20** | **1.9 (1.2-2.9)** | **8 (0-16)** |  | **8** | **1.4 (0.7-2.7)** | **2 (-3-7)** |  | **2** | **0.9 (0.2-3.5)** | **-1 (-7-5)** |
| Inflammatory diseases of the eye | 8 | 5.2 (2.6-10.5) | 6 (1-11) |  | 1 | 0.9 (0.1-6.2) | 0 (-2-2) |  | 0 | - | - |
| Other diseases and conditions of the eye | 7 | 1.2 (0.6-2.6) | 1 (-4-6) |  | 5 | 1.3 (0.5-3.2) | 1 (-3-5) |  | 2 | 1.3 (0.3-5.3) | 1 (-5-7) |
| Cataract | 5 | 1.5 (0.6-3.5) | 1 (-3-5) |  | 2 | 2.2 (0.6-8.9) | 1 (-1-3) |  | 0 | - | - |
| **Ear and mastoid** | **5** | **0.6 (0.3-1.5)** | **-3 (-6-1)** |  | **6** | **1.0 (0.5-2.3)** | **0 (-4-4)** |  | **3** | **1.3 (0.4-4.1)** | **2 (-6-9)** |
| Diseases of middle ear and mastoid process | 3 | 0.9 (0.3-2.9) | 0 (-3-3) |  | 3 | 1.1 (0.4-3.6) | 0 (-3-3) |  | 2 | 1.9 (0.5-7.6) | 2 (-4-8) |
| Diseases of inner ear | 1 | 0.3 (0.0-2.4) | -2 (-3-0) |  | 3 | 1.3 (0.4-3.9) | 1 (-2-4) |  | 0 | - | - |
| Other diseases of ear | 1 | 0.6 (0.1-4.3) | -1 (-2-1) |  | 0 | - | - |  | 1 | 2.9 (0.4-20.5) | 1 (-3-6) |
| **Circulatory** | **458** | **2.7 (2.5-3.0)** | **259 (221-297)** |  | **167** | **1.8 (1.6-2.1)** | **66 (44-88)** |  | **129** | **3.6 (3.1-4.3)** | **201 (153-249)** |
| Hypertensive disease | 19 | 2.2 (1.4-3.4) | 9 (2-17) |  | 3 | 0.5 (0.2-1.5) | -3 (-6-0) |  | 1 | 0.4 (0.1-3.0) | -3 (-7-1) |
| Angina pectoris | 46 | 2.6 (2.0-3.5) | 26 (14-39) |  | 18 | 1.8 (1.1-2.9) | 7 (0-14) |  | 16 | 4.3 (2.6-7.1) | 27 (10-45) |
| Acute myocardial infarction | 53 | 2.9 (2.2-3.8) | 32 (19-45) |  | 14 | 1.7 (1.0-2.9) | 5 (-1-12) |  | 15 | 4.7 (2.8-7.7) | 26 (9-42) |
| Chronic ischaemic heart disease | 35 | 3.5 (2.5-4.9) | 23 (12-33) |  | 13 | 3.0 (1.7-5.2) | 8 (1-14) |  | 10 | 6.0 (3.2-11.1) | 18 (5-32) |
| Pulmonary embolism | 8 | 3.2 (1.6-6.3) | 5 (0-10) |  | 1 | 0.6 (0.1-4.5) | -1 (-2-1) |  | 0 | - | - |
| Acute and chronic diseases of pericardium | 10 | 9.2 (4.9-17.3) | 8 (2-14) |  | 4 | 3.2 (1.2-8.6) | 2 (-1-6) |  | 2 | 3.9 (1.0-15.8) | 3 (-3-9) |
| Acute and subacute endocarditis | 11 | 23.4 (12.8-43.1) | 9 (4-15) |  | 0 | - | - |  | 3 | 18.3 (5.8-57.6) | 6 (-1-13) |

| Table S2. Continued |  | | |  |  | | | | | | |
| --- | --- | --- | --- | --- | --- | --- | --- | --- | --- | --- | --- |
|  | **Survivors of Hodgkin lymphoma diagnosed**  **1943-1976** | | |  | **Survivors of Hodgkin lymphoma diagnosed**  **1977-2004** | | | | | | |
|  | **Early mixed subcohort^a^** | | |  | **Primary treatment only subcohort^b^** | | |  | **Assumed relapse subcohort^c^** | | |
|  | **No. of new diagnoses**  **obs.** | **RR**  **(95% CI)** | **AER^d^**  **(95% CI)** |  | **No. of new diagnoses**  **obs.** | **RR**  **(95% CI)** | **AER^d^**  **(95% CI)** |  | **No. of new diagnoses obs.** | **RR**  **(95% CI)** | **AER^d^**  **(95% CI)** |
| Mitral valve disorders | 12 | 11.5 (6.5-20.4) | 10 (4-16) |  | 4 | 9.9 (3.7-26.9) | 3 (0-7) |  | 1 | 6.4 (0.9-45.7) | 2 (-2-6) |
| Aortic valve disorders | 44 | 24.7 (18.2-33.5) | 38 (27-50) |  | 14 | 18.4 (10.7-31.4) | 12 (5-18) |  | 10 | 34.9 (18.6-65.7) | 21 (8-34) |
| Cardiomyopathy | 7 | 4.9 (2.3-10.3) | 5 (0-10) |  | 3 | 2.5 (0.8-7.7) | 2 (-1-5) |  | 5 | 10.2 (4.2-24.6) | 10 (0-19) |
| Atrioventricular block and other conduction disorders | 30 | 4.7 (3.3-6.7) | 21 (12-31) |  | 5 | 3.1 (1.3-7.5) | 3 (-1-7) |  | 5 | 7.6 (3.2-18.4) | 9 (0-19) |
| Cardiac arrest | 4 | 5.1 (1.9-13.6) | 3 (-1-6) |  | 2 | 3.8 (1.0-15.4) | 1 (-1-4) |  | 0 | - | - |
| Paroxysmal tachycardia | 8 | 2.3 (1.1-4.6) | 4 (-1-9) |  | 5 | 1.4 (0.6-3.4) | 1 (-3-5) |  | 7 | 5.4 (2.6-11.4) | 12 (1-24) |
| Atrial fibrillation and flutter | 20 | 2.3 (1.5-3.6) | 10 (2-18) |  | 14 | 2.7 (1.6-4.6) | 8 (1-14) |  | 8 | 4.1 (2.0-8.1) | 13 (1-25) |
| Other cardiac arrhythmias | 9 | 5.3 (2.8-10.3) | 7 (1-12) |  | 1 | 0.7 (0.1-5.2) | 0 (-2-1) |  | 1 | 2.0 (0.3-14.0) | 1 (-3-5) |
| Heart failure | 36 | 7.1 (5.1-9.9) | 28 (17-39) |  | 14 | 6.2 (3.6-10.5) | 10 (4-17) |  | 3 | 3.4 (1.1-10.7) | 5 (-3-12) |
| Other heart diseases | 4 | 5.1 (1.9-13.7) | 3 (-1-6) |  | 1 | 1.6 (0.2-11.6) | 0 (-1-2) |  | 2 | 8.3 (2.1-33.5) | 4 (-2-10) |
| Intracranial haemorrhage | 7 | 1.6 (0.8-3.4) | 2 (-2-7) |  | 4 | 1.6 (0.6-4.2) | 1 (-2-5) |  | 4 | 4.1 (1.5-10.9) | 7 (-2-15) |
| Cerebral infarction | 12 | 1.6 (0.9-2.8) | 4 (-2-10) |  | 5 | 1.3 (0.6-3.2) | 1 (-3-5) |  | 4 | 2.8 (1.0-7.4) | 6 (-3-14) |
| Stroke NOS^d^ | 15 | 1.8 (1.1-3.1) | 6 (-1-13) |  | 4 | 1.3 (0.5-3.4) | 1 (-3-4) |  | 4 | 3.3 (1.3-8.9) | 6 (-2-14) |
| Other cerebrovascular diseases | 5 | 1.5 (0.6-3.5) | 1 (-3-5) |  | 0 | - | - |  | 0 | - | - |
| Atriosclerosis | 8 | 1.5 (0.8-3.0) | 2 (-3-7) |  | 1 | 0.6 (0.1-4.2) | -1 (-2-1) |  | 5 | 7.8 (3.2-18.9) | 9 (0-19) |
| Atrial embolism and thrombosis | 4 | 2.9 (1.1-7.8) | 2 (-1-6) |  | 1 | 2.0 (0.3-14.5) | 0 (-1-2) |  | 1 | 5.1 (0.7-36.6) | 2 (-2-6) |
| Other peripheral vascular diseases | 4 | 0.8 (0.3-2.1) | -1 (-5-3) |  | 0 | - | - |  | 0 | - | - |
| Phlebitis and thrombophlebitis | 12 | 2.1 (1.2-3.8) | 6 (0-12) |  | 7 | 1.5 (0.7-3.1) | 2 (-3-7) |  | 9 | 5.0 (2.6-9.6) | 16 (3-28) |
| Venous embolism and thrombosis | 19 | 2.0 (1.3-3.1) | 8 (1-16) |  | 10 | 1.4 (0.8-2.6) | 3 (-3-8) |  | 9 | 3.4 (1.8-6.5) | 14 (1-26) |
| Other disorders of circulatory system | 16 | 0.6 (0.4-1.0) | -9 (-17--2) |  | 19 | 1.3 (0.8-2.0) | 4 (-4-11) |  | 4 | 0.7 (0.3-1.8) | -4 (-13-4) |
| **Respiratory** | **171** | **2.6 (2.3-3.0)** | **95 (72-118)** |  | **98** | **2.0 (1.6-2.4)** | **42 (25-59)** |  | **103** | **5.3 (4.4-6.4)** | **179 (137-222)** |
| Acute upper respiratory infections | 6 | 2.4 (1.1-5.4) | 3 (-1-8) |  | 7 | 2.0 (1.0-4.3) | 3 (-1-8) |  | 7 | 5.0 (2.4-10.6) | 12 (1-23) |
| Diseases of vocal cords and larynx | 5 | 2.7 (1.1-6.5) | 3 (-1-7) |  | 2 | 1.5 (0.4-6.1) | 1 (-2-3) |  | 2 | 4.2 (1.0-16.7) | 3 (-3-9) |
| Other diseases of upper respiratory tract | 9 | 0.8 (0.4-1.6) | -2 (-7-4) |  | 8 | 0.6 (0.3-1.1) | -5 (-10--1) |  | 3 | 0.5 (0.2-1.5) | -7 (-14-1) |
| Pneumonia | 57 | 3.0 (2.3-3.9) | 35 (21-49) |  | 35 | 2.6 (1.9-3.7) | 19 (9-29) |  | 40 | 8.0 (5.8-10.9) | 78 (50-105) |
| Other acute lower respiratory infections | 4 | 1.7 (0.6-4.5) | 1 (-2-5) |  | 5 | 3.5 (1.5-8.5) | 3 (-1-7) |  | 8 | 15.7 (7.8-31.6) | 16 (4-28) |
| Bronchitis | 10 | 2.5 (1.4-4.7) | 5 (0-11) |  | 1 | 1.5 (0.2-10.6) | 0 (-1-2) |  | 2 | 7.8 (1.9-31.3) | 4 (-2-10) |
| Chronic obstructive pulmonary disease | 8 | 1.0 (0.5-2.1) | 0 (-5-5) |  | 3 | 1.1 (0.3-3.3) | 0 (-3-3) |  | 4 | 4.2 (1.6-11.3) | 7 (-2-15) |
| Asthma | 5 | 1.2 (0.5-2.8) | 1 (-3-5) |  | 2 | 0.6 (0.1-2.3) | -1 (-4-1) |  | 3 | 2.2 (0.7-6.8) | 3 (-4-11) |
| Lung diseases due to external agents | 3 | 8.9 (2.8-27.9) | 2 (-1-5) |  | 0 | - | - |  | 4 | 31.2 (11.5-84.7) | 8 (0-17) |
| Pulmonary oedema and other interstitial diseases | 10 | 8.2 (4.4-15.4) | 8 (2-13) |  | 4 | 8.0 (3.0-21.5) | 3 (0-6) |  | 5 | 27.3 (11.2-66.6) | 10 (1-20) |
| Abscess of lung and pleural empyema | 1 | 1.1 (0.1-7.5) | 0 (-2-2) |  | 7 | 7.8 (3.7-16.4) | 5 (1-10) |  | 3 | 8.2 (2.6-25.5) | 6 (-2-13) |
| Pneumothorax | 7 | 4.5 (2.1-9.5) | 5 (0-10) |  | 3 | 1.9 (0.6-5.9) | 1 (-2-4) |  | 5 | 7.7 (3.2-18.5) | 9 (0-19) |
| Pleural effusion and other pleural conditions | 19 | 10.5 (6.7-16.6) | 16 (8-23) |  | 8 | 7.7 (3.8-15.6) | 6 (1-11) |  | 5 | 12.8 (5.3-30.9) | 10 (0-19) |
| Respiratory failure | 19 | 5.7 (3.6-8.9) | 14 (6-22) |  | 8 | 3.5 (1.7-7.0) | 5 (0-10) |  | 8 | 9.9 (4.9-19.9) | 16 (4-27) |
| Other respiratory disorders | 8 | 2.5 (1.3-5.1) | 4 (-1-9) |  | 5 | 2.8 (1.2-6.7) | 3 (-1-7) |  | 4 | 5.6 (2.1-14.9) | 7 (-1-16) |
| **Digestive** | **188** | **1.6 (1.4-1.8)** | **61 (37-85)** |  | **88** | **0.9 (0.7-1.1)** | **-10 (-26-6)** |  | **87** | **2.2 (1.8-2.7)** | **101 (62-141)** |
| Diseases of oral cavity, salivary glands and jaws | 9 | 2.4 (1.2-4.6) | 5 (-1-10) |  | 5 | 1.3 (0.5-3.2) | 1 (-3-5) |  | 5 | 3.2 (1.3-7.7) | 7 (-2-17) |
| Oesophagitis and other diseases of oesophagus | 17 | 4.1 (2.5-6.6) | 12 (4-19) |  | 7 | 1.7 (0.8-3.6) | 2 (-2-7) |  | 4 | 2.4 (0.9-6.4) | 5 (-3-13) |

| Table S2. Continued |  | | |  |  | | | | | | |
| --- | --- | --- | --- | --- | --- | --- | --- | --- | --- | --- | --- |
|  | **Survivors of Hodgkin lymphoma diagnosed**  **1943-1976** | | |  | **Survivors of Hodgkin lymphoma diagnosed**  **1977-2004** | | | | | | |
|  | **Early mixed subcohort^a^** | | |  | **Primary treatment only subcohort^b^** | | |  | **Assumed relapse subcohort^c^** | | |
|  | **No. of new diagnoses**  **obs.** | **RR**  **(95% CI)** | **AER^d^**  **(95% CI)** |  | **No. of new diagnoses**  **obs.** | **RR**  **(95% CI)** | **AER^d^**  **(95% CI)** |  | **No. of new diagnoses obs.** | **RR**  **(95% CI)** | **AER^d^**  **(95% CI)** |
| Peptic ulcer of stomach and duodenum | 18 | 1.6 (1.0-2.6) | 6 (-1-14) |  | 8 | 1.5 (0.8-3.1) | 2 (-2-7) |  | 7 | 3.3 (1.6-7.0) | 11 (-1-22) |
| Other diseases of stomach and duodenum | 13 | 1.4 (0.8-2.4) | 3 (-3-10) |  | 9 | 1.4 (0.7-2.7) | 2 (-3-7) |  | 8 | 3.1 (1.5-6.2) | 12 (0-24) |
| Hernia | 19 | 0.8 (0.5-1.3) | -4 (-12-4) |  | 14 | 1.0 (0.6-1.6) | -1 (-7-6) |  | 6 | 1.0 (0.4-2.2) | 0 (-11-10) |
| Crohn disease and ulcerative colitis | 3 | 1.2 (0.4-3.7) | 0 (-3-3) |  | 1 | 0.3 (0.0-2.0) | -2 (-4--1) |  | 0 | - | - |
| Other noninfective gastroenteritis and colitis | 4 | 2.2 (0.8-5.9) | 2 (-2-5) |  | 0 | - | - |  | 4 | 5.3 (2.0-14.1) | 7 (-1-15) |
| Paralytic ileus without hernia | 21 | 5.4 (3.5-8.3) | 16 (7-24) |  | 5 | 2.0 (0.8-4.8) | 2 (-2-6) |  | 8 | 8.5 (4.2-17.1) | 15 (3-27) |
| Other functional intestinal disorders | 17 | 1.9 (1.2-3.1) | 7 (0-15) |  | 3 | 0.4 (0.1-1.3) | -3 (-6-0) |  | 5 | 1.9 (0.8-4.6) | 5 (-4-15) |
| Fissure, fistula and abscess of anal and rectal regions | 14 | 1.6 (0.9-2.6) | 5 (-2-11) |  | 6 | 0.7 (0.3-1.5) | -3 (-7-2) |  | 6 | 1.6 (0.7-3.6) | 5 (-5-15) |
| Other diseases of intestine | 10 | 1.0 (0.5-1.8) | 0 (-6-5) |  | 7 | 0.6 (0.3-1.4) | -3 (-8-1) |  | 10 | 2.2 (1.2-4.1) | 12 (-2-25) |
| Acute peritonitis | 3 | 2.6 (0.8-8.1) | 2 (-1-5) |  | 3 | 2.9 (0.9-9.0) | 2 (-1-5) |  | 3 | 7.5 (2.4-23.3) | 6 (-2-13) |
| Hepatic failure | 3 | 1.1 (0.4-3.5) | 0 (-3-3) |  | 1 | 0.8 (0.1-5.7) | 0 (-2-1) |  | 1 | 1.9 (0.3-13.6) | 1 (-3-5) |
| Other diseases of liver | 2 | 0.5 (0.1-2.2) | -2 (-4-1) |  | 3 | 0.8 (0.3-2.4) | -1 (-4-2) |  | 1 | 0.7 (0.1-4.8) | -1 (-5-3) |
| Cholelithiasis | 10 | 0.8 (0.4-1.5) | -2 (-8-4) |  | 11 | 0.7 (0.4-1.3) | -3 (-9-2) |  | 10 | 1.9 (1.0-3.5) | 10 (-3-24) |
| Acute pancreatitis | 5 | 1.4 (0.6-3.4) | 1 (-3-5) |  | 0 | - | - |  | 2 | 1.6 (0.4-6.3) | 2 (-4-8) |
| Other disease of digestive system | 20 | 2.5 (1.6-3.9) | 11 (3-19) |  | 5 | 0.8 (0.3-1.8) | -1 (-5-2) |  | 7 | 2.7 (1.3-5.7) | 10 (-2-21) |
| **Skin and subcutaneous tissue** | **32** | **1.7 (1.2-2.4)** | **12 (2-22)** |  | **28** | **1.3 (0.9-1.9)** | **6 (-4-15)** |  | **20** | **2.2 (1.4-3.5)** | **24 (5-43)** |
| Cutaneous abscess, furuncle and carbuncle | 3 | 0.7 (0.2-2.2) | -1 (-4-2) |  | 9 | 1.2 (0.6-2.3) | 1 (-4-6) |  | 7 | 2.3 (1.1-4.8) | 9 (-3-20) |
| Cellulitis | 3 | 1.5 (0.5-4.7) | 1 (-2-4) |  | 6 | 2.8 (1.3-6.2) | 3 (-1-8) |  | 2 | 2.2 (0.5-8.7) | 2 (-4-8) |
| Pyoderma and erythrasma | 12 | 3.2 (1.8-5.7) | 8 (1-14) |  | 3 | 1.5 (0.5-4.7) | 1 (-2-4) |  | 1 | 1.1 (0.2-8.0) | 0 (-4-4) |
| Other infections of skin and subcutaneous tissue | 0 | - | - |  | 2 | 0.7 (0.2-2.9) | -1 (-3-2) |  | 1 | 0.9 (0.1-6.1) | 0 (-5-4) |
| Other diseases of skin and subcutaneous tissue | 14 | 1.7 (1.0-2.9) | 5 (-1-12) |  | 8 | 1.1 (0.6-2.2) | 1 (-4-6) |  | 9 | 3.1 (1.6-6.0) | 13 (1-26) |
| **Musculoskeletal and connective tissue** | **81** | **1.1 (0.9-1.3)** | **4 (-11-20)** |  | **56** | **0.8 (0.6-1.0)** | **-14 (-27--1)** |  | **42** | **1.5 (1.1-2.0)** | **28 (1-55)** |
| Infectious arthropathies | 1 | 1.2 (0.2-8.4) | 0 (-2-2) |  | 2 | 1.4 (0.3-5.4) | 0 (-2-3) |  | 1 | 1.6 (0.2-11.4) | 1 (-3-5) |
| Rheumatoid arthritis | 1 | 0.4 (0.1-2.6) | -2 (-3-0) |  | 0 | - | - |  | 0 | - | - |
| Arthrosis | 15 | 1.4 (0.9-2.4) | 4 (-3-11) |  | 4 | 0.3 (0.1-0.8) | -7 (-11--4) |  | 4 | 0.8 (0.3-2.2) | -2 (-10-6) |
| Systemic connective tissue disorders | 9 | 1.3 (0.7-2.4) | 2 (-4-7) |  | 2 | 0.7 (0.2-2.8) | -1 (-3-2) |  | 2 | 1.7 (0.4-6.8) | 2 (-4-8) |
| Kyphosis, lordosis and other deforming dorsopathies | 2 | 3.3 (0.8-13.3) | 1 (-1-4) |  | 2 | 2.1 (0.5-8.5) | 1 (-1-3) |  | 1 | 2.8 (0.4-20.0) | 1 (-3-6) |
| Spondylopathies | 5 | 1.1 (0.5-2.6) | 0 (-4-4) |  | 3 | 0.8 (0.2-2.3) | -1 (-4-2) |  | 4 | 2.8 (1.0-7.3) | 6 (-3-14) |
| Intervertebral disc disorders | 10 | 0.6 (0.3-1.1) | -7 (-13--1) |  | 15 | 0.9 (0.6-1.5) | -1 (-8-6) |  | 7 | 1.1 (0.5-2.2) | 1 (-10-12) |
| Other dorsopathies | 4 | 0.6 (0.2-1.7) | -2 (-6-1) |  | 5 | 0.6 (0.3-1.5) | -2 (-6-1) |  | 7 | 2.3 (1.1-4.7) | 8 (-3-20) |
| Disorders of muscles | 6 | 1.7 (0.8-3.8) | 2 (-2-7) |  | 5 | 1.6 (0.7-3.8) | 2 (-2-5) |  | 2 | 1.6 (0.4-6.3) | 2 (-4-8) |
| Other soft tissue disorders | 15 | 1.2 (0.7-2.0) | 2 (-5-9) |  | 12 | 0.8 (0.4-1.4) | -3 (-9-3) |  | 5 | 0.8 (0.3-2.0) | -2 (-12-7) |
| Osteopathies and chondropathies | 5 | 1.8 (0.8-4.4) | 2 (-2-6) |  | 4 | 1.5 (0.6-4.1) | 1 (-2-5) |  | 6 | 5.7 (2.6-12.7) | 11 (0-21) |
| Other disorders of musculoskeletal system and connective tissue | 8 | 1.1 (0.6-2.3) | 1 (-4-6) |  | 2 | 0.5 (0.1-1.9) | -2 (-4-1) |  | 3 | 1.7 (0.5-5.2) | 3 (-5-10) |
| **Urinary system and genital organs** | **118** | **1.1 (0.9-1.3)** | **10 (-9-29)** |  | **86** | **1.1 (0.9-1.4)** | **7 (-9-22)** |  | **54** | **1.8 (1.4-2.4)** | **53 (22-84)** |
| Glomerular diseases | 2 | 2.2 (0.5-8.7) | 1 (-2-3) |  | 2 | 3.1 (0.8-12.6) | 1 (-1-4) |  | 2 | 7.8 (1.9-31.2) | 4 (-2-10) |
| Tubulointestinal nephritis | 4 | 1.6 (0.6-4.4) | 1 (-2-5) |  | 5 | 2.2 (0.9-5.3) | 2 (-1-6) |  | 4 | 5.0 (1.9-13.3) | 7 (-2-15) |
| Obstructive and reflux uropathy | 6 | 4.6 (2.0-10.2) | 4 (0-9) |  | 1 | 0.8 (0.1-5.7) | 0 (-2-1) |  | 0 | - | - |
| Renal failure | 9 | 4.0 (2.1-7.7) | 6 (1-11) |  | 3 | 1.9 (0.6-6.0) | 1 (-2-4) |  | 5 | 8.4 (3.5-20.2) | 9 (0-19) |

| Table S2. Continued |  | | |  |  | | | | | | |
| --- | --- | --- | --- | --- | --- | --- | --- | --- | --- | --- | --- |
|  | **Survivors of Hodgkin lymphoma diagnosed**  **1943-1976** | | |  | **Survivors of Hodgkin lymphoma diagnosed**  **1977-2004** | | | | | | |
|  | **Early mixed subcohort^a^** | | |  | **Primary treatment only subcohort^b^** | | |  | **Assumed relapse subcohort^c^** | | |
|  | **No. of new diagnoses**  **obs.** | **RR**  **(95% CI)** | **AER^d^**  **(95% CI)** |  | **No. of new diagnoses**  **obs.** | **RR**  **(95% CI)** | **AER^d^**  **(95% CI)** |  | **No. of new diagnoses obs.** | **RR**  **(95% CI)** | **AER^d^**  **(95% CI)** |
| Ureterolithiasis | 11 | 1.4 (0.8-2.6) | 3 (-3-9) |  | 5 | 0.7 (0.3-1.7) | -2 (-6-2) |  | 5 | 1.7 (0.7-4.1) | 4 (-5-14) |
| Other disorders of kidney | 1 | 1.0 (0.1-7.2) | 0 (-2-2) |  | 2 | 5.5 (1.3-22.0) | 1 (-1-4) |  | 1 | 6.7 (0.9-47.7) | 2 (-2-6) |
| Cystitis | 6 | 1.1 (0.5-2.5) | 1 (-4-5) |  | 3 | 0.9 (0.3-2.7) | 0 (-3-3) |  | 4 | 3.2 (1.2-8.6) | 6 (-3-14) |
| Urethral stricture | 1 | 0.7 (0.1-5.2) | 0 (-2-1) |  | 3 | 4.5 (1.4-13.9) | 2 (-1-5) |  | 2 | 6.7 (1.7-27.0) | 4 (-2-10) |
| Other disorders of urinary system | 6 | 1.2 (0.5-2.7) | 1 (-3-5) |  | 6 | 1.7 (0.7-3.7) | 2 (-2-6) |  | 1 | 0.8 (0.1-5.6) | -1 (-5-4) |
| Hyperplasia of prostate | 6 | 1.0 (0.4-2.2) | 0 (-4-4) |  | 2 | 1.3 (0.3-5.4) | 0 (-2-3) |  | 1 | 1.8 (0.3-12.7) | 1 (-3-5) |
| Other diseases of male genital organs | 12 | 1.5 (0.8-2.6) | 4 (-3-10) |  | 7 | 1.2 (0.6-2.4) | 1 (-4-5) |  | 7 | 2.6 (1.2-5.4) | 9 (-2-21) |
| Disorders of breast | 6 | 0.8 (0.3-1.7) | -2 (-6-3) |  | 5 | 0.9 (0.4-2.2) | 0 (-4-3) |  | 2 | 1.0 (0.3-4.0) | 0 (-6-6) |
| Inflammatory diseases of female genital organs | 6 | 0.7 (0.3-1.6) | -2 (-6-2) |  | 5 | 0.6 (0.2-1.3) | -3 (-7-0) |  | 4 | 1.2 (0.5-3.3) | 2 (-7-10) |
| Endometriosis | 2 | 1.3 (0.3-5.2) | 0 (-2-3) |  | 1 | 0.4 (0.1-2.6) | -2 (-3-0) |  | 1 | 1.0 (0.1-7.2) | 0 (-4-4) |
| Other non–inflammatory disorders of female genital organs | 40 | 0.9 (0.6-1.2) | -6 (-18-6) |  | 35 | 1.1 (0.8-1.5) | 3 (-8-13) |  | 15 | 1.3 (0.8-2.2) | 8 (-9-24) |
| Other disorders of genitourinary system | 0 | - | - |  | 1 | 2.4 (0.3-16.9) | 1 (-1-2) |  | 0 | - | - |
| **Special calculations for infections** |  |  |  |  |  |  |  |  |  |  |  |
| Infections classified in other ICD-chapters^e^ | 109 | 2.5 (2.0-3.0) | 58 (40-76) |  | 84 | 2.2 (1.8-2.7) | 40 (24-55) |  | 83 | 5.5 (4.4-6.8) | 146 (107-184) |
| All infections^f^ | 172 | 2.5 (2.2-2.9) | 93 (70-116) |  | 137 | 2.2 (1.8-2.6) | 64 (44-84) |  | 133 | 5.3 (4.5-6.3) | 232 (183-280) |
| ^a^ No information on re-hospitalisations available.  ^b^ No re-hospitalisation with Hodgkin lymphoma as primary diagnosis in an oncological or haematological department one year or more after the first Hodgkin lymphoma diagnosis or, if re-hospitalised for Hodgkin lymphoma 6 months to one year after the first Hodgkin lymphoma diagnosis, did not die within 10 years after the first Hodgkin lymphoma diagnosis.  ^c^ Re-hospitalisation with Hodgkin lymphoma as primary diagnosis in an oncologic or haematologic department one year or more after the first Hodgkin lymphoma diagnosis, or re-hospitalisation 6 months to one year after the first Hodgkin lymphoma diagnosis and deceased within 10 years of the first Hodgkin lymphoma diagnosis.  ^d^ Absolute excess rate per 10,000 person-years; the rates are standardised for age, sex and calendar periods.  ^e^ From **Nervous system and sense organs**: bacterial meningitis, encephalitis, myelitis and encephalomyelitis and intracranial and intraspinal abscess; from **Circulatory:** Acute and subacute endocarditis; from **Respiratory**: Acute upper respiratory infections, pneumonia, other acute lower respiratory infections, bronchitis, abscess of lung and pleural empyema; from **Digestive:** acute peritonitis; from **Skin and subcutaneous**: cutaneous abscess, furuncle and carbuncle, cellulitis, other infections of skin and subcutaneous tissue; from **Musculoskeletal and connective tissue**: infectious arthropathies, from **Urinary and genital**: cystitis.  ^f^ The sum of infections classified in the ICD chapter ‘Infections’ and infections classified in other ICD chapters. | | | | | | | | | | | |
